# Supplementary material for: Origins of glycan selectivity in streptococcal Siglec-like adhesins suggest mechanisms of receptor adaptation
Source: Nat Commun. 2022 May 18;13:2753. doi: 10.1038/s41467-022-30509-y (PMC9117288; doi:10.1038/s41467-022-30509-y)
Supplement: Supplementary file 1 — Supplementary Info [file 41467_2022_30509_MOESM1_ESM.pdf]

Supplementary Information
Origins of glycan selectivity in streptococcal Siglec-like adhesins suggest mechanisms of receptor adaptation

AUTHORS

Barbara A. Bensing[1,$], Haley E. Stubbs[2,$], Rupesh Agarwal[3,4], Izumi Yamakawa[5,&], Kelvin Luong[5], Kemal Solakyildirim[6,7], Hai Yu[7], Azadeh Hadadianpour[8], Manuel A Castro[9], Kevin P. Fialkowski[5,†], KeAndreya M. Morrison[10], Zdzislaw Wawrzak[11], Xi Chen[7], Carlito B. Lebrilla[7], Jerome Baudry[12], Jeremy C. Smith[3,4,13], Paul M. Sullam[1], T M Iverson[5,9,*]

[1]Division of Infectious Diseases, Veterans Affairs Medical Center, Department of Medicine, University of California, San Francisco, and the Northern California Institute for Research and Education, San Francisco, California 94121, USA
[2]Graduate Program in Chemical and Physical Biology, Vanderbilt University, Nashville, Tennessee 37232, USA
[3]University of Tennessee/Oak Ridge National Laboratory, Center for Molecular Biophysics, Biosciences Division, Oak Ridge National Laboratory, Oak Ridge, Tennessee 37831-6309, USA
[4]Department of Biochemistry and Cellular and Molecular Biology, University of Tennessee, Knoxville, Tennessee, 37996, USA
[5]Department of Pharmacology, Vanderbilt University, Nashville, Tennessee 37232, USA
[6]Department of Chemistry, Erzincan Binali Yildirim University, Erzincan, 24100, Turkey
[7]Department of Chemistry, University of California, Davis, California, 95616, USA
[8]Department of Microbiology, Pathology, and Immunology, Vanderbilt University, Nashville, Tennessee, 37232, USA
[9]Department of Biochemistry, Vanderbilt University, Nashville, Tennessee, 37232, USA
[10]Department of Pharmacology, School of Graduate Studies and Research, Meharry Medical College, Nashville, TN, 37208
[11]LS-CAT Synchrotron Research Center, Northwestern University, Argonne, Illinois, 60439, USA
[12]Department of Biological Sciences, The University of Alabama in Huntsville, Huntsville, Alabama 35899, USA
[13]Department of Biochemistry and Cellular and Molecular Biology, University of Tennessee, Knoxville, Tennessee, 37996

[$]These authors contributed equally to this manuscript.
†Present address: College of Medicine, University of Arkansas for Medical Sciences, Little Rock, AR 72205, USA
[&]Present address: School of Nursing, Belmont University, Nashville, TN 37212, USA
*To whom correspondence may be addressed: (tina.iverson@vanderbilt.edu).

Supplementary Table 1

	$SLBR_{Hsa}$	$SLBR_{UB10712}$	$SLBR_{SK678}$
PDB entry	6EFC	6EFF	6EFI
DATAID	328	509	510
Resolution	1.4 Å	1.6 Å	1.7 Å
Data collection			
Beamline	APS 21-ID-F	APS 21-ID-F	SSRL 9-2
Wavelength	0.978 Å	0.978 Å	0.979 Å
Space group	$P2_12_12_1$	P1	$P2_1$
Unit cell	a=46.6 Å<br>b=58.1 Å<br>c=76.0 Å	a=39.8 Å<br>b=48.9 Å<br>c=99.8 Å<br>α=101.8°<br>β=91.4°<br>γ=89.9°	a=59.6 Å<br>b=59.58 Å<br>c=61.8 Å<br>β=100.7°
R_{sym}	0.084 (0.650)	0.075 (0.730)	0.099 (0.530)
R_{pim}	0.024 (0.281)	0.047 (0.479)	0.040 (0.213)
I/σ	49.7 (2.3)	22.9 (1.9)	15.0 (4.4)
Completeness (%)	93.3% (60.9%)	92.4% (70.9%)	97.7% (97.3%)
Redundancy	12.6 (5.6)	3.6 (3.4)	7.0 (7.1)
$CC_{1/2}$	0.837	0.648	0.998
Refinement			
R_{cryst}	0.146[&]	0.180	0.177
R_{free}	0.179	0.207	0.210
No. Mol per ASU	1	4	2
RMS deviation			
bond lengths	0.01 Å	0.01 Å	0.01 Å
bond angles	1.6°	0.9°	0.7°
Ramachandran			
favored	97.0%	96.8%	99.0%
allowed	2.5%	3.1%	1.0%
outliers	0.5%*	0.1%	0.0%

Supplementary Table 1. Crystallographic data collection and refinement statistics for unliganded $SLBR_{Hsa}$, $SLBR_{UB10712}$, and $SLBR_{SK678}$. Values in parentheses are for the highest resolution shell. Raw data are deposited with SBGrid and can be accessed at: data.sbgrid.org/dataset/DATAID. The Ramachandran angles identified as outliers ($SLBR_{Hsa}^{S253}$, $SLBR_{Hsa}^{L363}$, $SLBR_{UB10712}^{S253}$, $SLBR_{UB10712}^{L361}$) are associated with clear electron density. Data collected at the Advanced Photon Source (APS) used the Remote Access, data collected at Stanford Synchrotron Radiation Lightsource (SSRL) used the Blu-Ice software.

Supplementary Table 2

	$SLBR_{GspB\text{-}Siglec}$ Form 1	$SLBR_{GspB\text{-}Siglec}$ Form 2	$SLBR_{GspB}$	$SLBR_{SK150}$
PDB entry	6EF7	6EF9	6EFA	6EFB
SBGrid DATAID	812	601	604	508
Resolution	1.03 Å	1.3 Å	1.6 Å	1.90 Å
Data collection				
Beamline	21-ID-F	21-ID-G	21-ID-F	Bruker X8R
Wavelength	0.979 Å	0.979 Å	0.979 Å	1.542 Å
Space group	$P2_12_12$	R32	$P2_12_12_1$	$P2_1$
Unit cell	a= 33.9 Å	a=b=92.1 Å	a=33.0 Å	a=24.3 Å
	b= 46.2 Å		b=47.6 Å	b=62.6 Å
	c= 73.0 Å	c=143.9 Å	c=136.2 Å	c=62.9 Å
				β=98.6°
R_{sym}	0.049 (0.430)	0.061 (0.771)	0.057 (0.610)	0.139 (0.538)
R_{pim}	0.024 (0.233)	0.017 (0.302)	0.019 (0.213)	0.044 (0.295)
I/σ	25.5 (4.4)	59.3 (2.8)	43.8 (3.3)	9.3 (1.9)
Completeness (%)	95.4% (90.6%)	99.9% (98.0%)	88.9% (48.6%)	97.3% (91.6%)
Redundancy	9.3 (8.2)	13.8 (7.1)	9.2 (7.7)	9.0 (3.6)
$CC_{1/2}$	0.911	0.941	0.975	0.996
Refinement				
R_{cryst}	0.125	0.131	0.166	0.172
R_{free}	0.141	0.144	0.209	0.188
No. Mol per ASU	1	2	1	1
RMS deviation				
bond lengths	0.01 Å	0.01 Å	0.02 Å	0.01 Å
bond angles	1.3°	1.1°	1.6°	0.7°
Ramachandran				
favored	100%	97.0%	98.0%	99.0%
allowed	0%	2.2%	1.5%	1.0%
outliers*	0%	0.8%	0.5%	0.0%

Supplementary Table 2. Crystallographic data collection and refinement statistics for unliganded $SLBR_{GspB}$, $SLBR_{GspB\text{-}Siglec}$ and $SLBR_{SK150}$. Values in parentheses are for the highest resolution shell. Raw data are deposited with SBGrid and can be accessed at: data.sbgrid.org/dataset/DATAID. Data collected at the APS used the remote access software, data collected on the Bruker X8R used the CR-XRD software.

Supplementary Table 3

	**$SLBR_{Hsa}$ + sTa**	**$SLBR_{Hsa}$ + 3'sLn**	**$SLBR_{Hsa}$ + 6S-sLe^X**	**$SLBR_{Hsa}$ + sLe^C**	**$SLBR_{GspB-Siglec}$ + sTa**
PDB entry	6EFD	6X3Q	6X3K	7KMJ	5IUC
DATAID	329	788	787	813	507
Resolution	1.85 Å	2.2 Å	2.47 Å	1.3 Å	1.25Å
Data collection					
Beamline	APS 21-ID-G	SSRL 9-2	SSRL 9-2	SSRL 9-2	21-ID-F
Wavelength	0.978 Å	0.979 Å	0.979 Å	0.979 Å	0.979 Å
Space group	$P2_12_12_1$	$P2_12_12_1$	$P2_12_12_1$	$P2_12_12_1$	$P2_12_12$
Unit cell	a=46.7 Å<br>b= 58.0 Å<br>c=76.1 Å	a=44.9 Å<br>b=57.1 Å<br>c=76.3 Å	a=47.7 Å<br>b=57.8 Å<br>c=75.7 Å	a=46.6 Å<br>b=58.1 Å<br>c=76.0 Å	a=67.7 Å<br>b=66.6 Å<br>c=55.9 Å
R_{sym}	0.107 (0.638)	0.126 (0.643)	0.123 (0.740)	0.076 (0.696)	0.066 (0.406)
R_{pim}	0.037 (0.218)	0.055 (0.283)	0.053 (0.318)	0.025 (0.263)	0.018 (0.111)
I/σ	31.7 (2.9)	12.1 (1.5)	15.6 (1.7)	40.5 (1.6)	35.6 (7.9)
Completeness (%)	98.8 % (89.5%)	99.6% (96.4%)	98.7% (99.7%)	99.7% (98.5%)	95.7% (90.5%)
Redundancy	9.5 (8.7)	4.6 (4.8)	4.9 (5.1)	8.0 (6.7)	14.9 (14.3)
$CC_{1/2}$	0.940	0.993	0.989	0.998	0.964
Refinement					
R_{cryst}	0.196[&]	0.206	0.236	0.187	0.156
R_{free}	0.217	0.233	0.250	0.216	0.178
No. Mol per ASU	1	1	1	1	2
RMS deviation					
bond lengths	0.01 Å	0.02 Å	0.03 Å	0.01 Å	0.01 Å
bond angles	0.9°	2.4°	2.1°	1.63°	1.5°
Ramachandran					
favored	97.1%	95.1%	95.5%	97.0%	99.2%
allowed	2.9%	4.4%	4.5%	2.5%	0.8%
outliers	0.0%[*]	0.5%	0.0%	0.5%	0.0%

Supplementary Table 3. Crystallographic data collection and refinement statistics for $SLBR_{Hsa}$ and $SLBR_{GspB-Siglec}$ bound to sialoglycans. Values in parentheses are for the highest resolution shell. Raw data are deposited with SBGrid and can be accessed at: data.sbgrid.org/dataset/DATAID. Data collected at the APS used the remote access software, data collected at SSRL used the Blu-Ice software.

Supplementary Table 4

	sTa	sLe^{C}	3'sLn	sLe^{X}	6S-sLe^{X}
**GST-SLBR$_{Hsa}$[a]**	0.12 ± 0.06	0.67 ± 0.18	1.61 ± 1.44	>5	>5
**E286R[a]**	0.11 ± 0.08	0.06 ± 0.08	0.11 ± 0.10	0.30 ± 0.17	0.01 ± 0.12[c]
**D356Q[a]**	0.55 ± 0.22	0.81 ± 0.52	0.17 ± 0.05	0.68 ± 0.36	0.00 ± 0.04[c]
**D356R[a]**	1.93 ± 2.21	0.74 ± 1.40	0.26 ± 0.09	2.62 ± 5.12	0.22 ± 0.05
**GST-SLBR$_{UB10712}$[a]**	>5	>5	0.13 ± 0.19	0.63 ± 0.18	0.14 ± 0.10
**E285R[a]**	>5	>5	1.40 ± 0.91	>5	0.00 ± 0.05[c]
**Q354D[a]**	1.89 ± 1.04	>5	0.58 ± 0.41	>5	>5
**+ SLBR$_{Hsa}$ CD loop[b]**	+	-	++	+	+++
**+ SLBR$_{Hsa}$ EF loop[b]**	+	+	+++	+++	+++
**+ SLBR$_{Hsa}$ FG loop[b]**	++	+	++	+	+
**+ all SLBR$_{Hsa}$ loops[a]**	0.17 ± 0.08	1.28 ± 2.36	>5	>5	>5
**GST-SLBR$_{SK678}$[a]**	>5	>5	0.90 ± 0.51	>5	0.63 ± 0.36
**E298R**	>5	>5	2.43 ± 1.66	>5	0.07 ± 0.09
**Q367D[a]**	>5	>5	>5	>5	>5
**+ SLBR$_{Hsa}$ CD loop[b]**	-	-	-	-	-
**+ SLBR$_{Hsa}$ EF loop[b]**	-	-	++	+	++
**+ SLBR$_{Hsa}$ FG loop[b]**	-	-	+	-	-
**+ all SLBR$_{Hsa}$ loops[a]**	0.67 ± 0.78	>5	>5	>5	>5
**GST-SLBR$_{GspB}$[a]**	0.08 ± 0.05	>5	>5	nd	nd
**L442Y/Y443N[a]**	0.31 ± 0.17	>5	0.15 ± 0.07	nd	nd
**+ SLBR$_{SK150}$ CD loop[b]**	-	nd	-	nd	nd
**+ SLBR$_{SK150}$ EF loop[b]**	+++	nd	-	nd	nd
**+ SLBR$_{SK150}$ FG loop[b]**	-	nd	-	nd	nd
**+ all SLBR$_{SK150}$ loops[b]**	-	nd	-	nd	nd
**GST-SLBR$_{SK150}$[a]**	1.09 ± 0.32	4.74 ± 6.31	2.91 ± 3.59	>5	>5
**Y300L/N301Y[a]**	>5	nd	>5	nd	nd

Supplementary Table 4. Summary of binding preferences of wild-type and variant SLBRs. Numbers reflect EC_{50} values while +/- designations are indicators of relative binding strength from one-point analysis. Abbreviations: sTa, sialyl T antigen; 3'sLn, 3'-sialyl-*N*-acetyllactosamine; sLe^{C}, sialyl Lewis^{C}; sLe^{X}, sialyl Lewis^{X}; 6S-sLe^{X}, 6-*O*-sulfo-sialyl Lewis^{X}; nd= not determined. Source data are provided as a Source Data file.

[a]EC_{50} values (µg/ml) ± standard errors were obtained via nonlinear regression of the ELISA curves in Figs. 6-8, and Supplementary Figures 1, 15, 17, and 19, using Prism 7 (GraphPad).

[b]Relative binding strengths are based on absorbance values obtained using 1-2 µg/ml biotinylated glycans. +++, $A_{450} > 1$; ++, $A_{450} = 0.7$-1; +, $A_{450} = 0.3$-0.7; -, $A_{450} < 0.3$.

[c]Approximate value

Supplementary Table 5

SLBR	Protein solution	Crystallization Reservoir Conditions	Cryo protectant	Data collection temp (°C)	Space group	Starting Model
$SLBR_{GspB}$	9 mg/mL in 20 mM Tris-HCl, pH 7.6	0.2 M $(NH_4)_2SO_4$, 25% polyethylene glycol (PEG) 3350	none	-180 °C	$P2_12_12_1$	3QC5[1]
$SLBR_{GspB-Siglec}$ Crystal form 1	22.8 mg/ml in 20 mM Tris-HCl, pH 7.2	0.2 M $MgCl_2$, 0.1 M Tris-HCl, pH 8.5, 30% w/v PEG 4000	25% glycerol	-180 °C	$P2_12_12$	3QC5[1] Siglec domain
$SLBR_{GspB-Siglec}$ Crystal form 1 + sTa	20.5 mg/ml in 10 mM sTa, 18 mM Tris-HCl, pH 7.2	0.2 M $MgCl_2$, 0.1 M Tris-HCl, pH 8.5, 30% w/v PEG 4000	25% glycerol	-180 °C	$P2_12_12$	Unliganded $SLBR_{GspB-Siglec}$
$SLBR_{GspB-Siglec}$ Crystal form 2	22.8 mg/ml in 20 mM Tris-HCl, pH 7.2	4.0 M HCOONa	none	-180 °C	R32	3QC5[1] Siglec domain
$SLBR_{SK150}$	3.5 mg/ml in 20 mM Tris-HCl, pH 7.6	0.2 M ammonium sulfate, 25% PEG 4000, 15% ethanol, and 0.1 M Bis-tris, pH 7.0	none	23 °C	$P2_1$	3QC5[1]
$SLBR_{Hsa}$	21.6 mg/ml in 20 mM Tris-HCl, pH 7.2	0.1 M Succinate/Phosphate/Glycine pH 10.0 and 25% PEG 3350	none	-180 °C	$P2_12_12_1$	5EQ2[2]
$SLBR_{Hsa}$ + sialoglycans	21.6 mg/ml in 20 mM Tris-HCl, pH 7.2	0.1 M Succinate/Phosphate/Glycine pH 10.0 and 25% PEG 3350	5 mM sialoglycan, 20 hr	-180 °C	$P2_12_12_1$	Unliganded $SLBR_{Hsa}$
$SLBR_{UB10712}$	3.5 mg/ml in 20 mM Tris-HCl pH 7.5	0.1 M Tris-HCl pH 7.5 and 32% w/v PEG 4000, with microseeding	50% glycerol	-180 °C	P1	Unliganded $SLBR_{Hsa}$
$SLBR_{SK678}$	7 mg/ml in 20 mM Tris-HCl pH 7.6	0.1 M Bicine pH 7.6 and 25% PEG 6,000, 0.005M hexamine cobalt(II) chloride	15% glycerol, 15% ethylene glycol	-180 °C	$P2_1$	$SLBR_{UB10712}$

Supplementary Table 5. Crystallization of SLBRs. SLBRs or $SLBR_{GspB-Siglec}$ were crystallized by the vapor diffusion method by equilibrating 1 µl protein and 1 µl reservoir solution over 50 µL – 1000 µL of the reservoir solution at room temperature. For data collected at -180 °C, crystals were cryo cooled by plunging in liquid nitrogen. Diffraction data were collected at the X-ray sources indicated **in Supplementary Tables 1 - 3**. Data for $SLBR_{GspB}$, $SLBR_{GspB-Siglec}$, and $SLBR_{Hsa}$ were processed with HKL2000 version 712 [3], data for $SLBR_{SK150}$ were processed using the PROTEUM suite, and data for $SLBR_{UB10712}$ and $SLBR_{SK678}$ were processed using XDS version June 1, 2017[4]. The structures were determined in PHENIX 1.18.2[5] using the indicated search models for molecular replacement. Structures of sialoglycan-bound $SLBR_{Hsa}$ were determined by rigid body refinement of unliganded $SLBR_{Hsa}$ in PHENIX 1.18.2[5] following the selection of the same set of R_{free} reflections.

Supplementary Figure 1

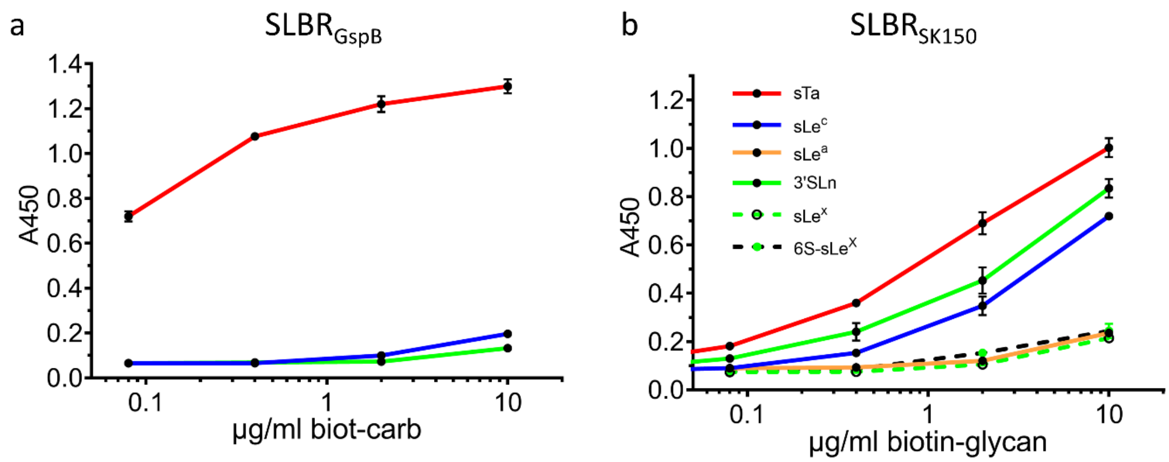

Supplementary Figure 1. Binding properties of $SLBR_{GspB}$ and $SLBR_{SK150}$. Dose response curves of biotin-glycan binding to immobilized **a** GST-$SLBR_{GspB}$ and **b** GST-$SLBR_{SK150}$. Measurements were performed by ELISA using 500 nM of immobilized GST-SLBR and the indicated concentrations of each ligand are shown as the mean ± SD (n = 3 independent experiments with a single protein preparation). sTa, sLe^C, and 3'sLn are trisaccharides; sLe^A, sLe^X, and 6S-sLe^X, are tetrasaccharides (Fig 1). Source data are provided as a Source Data file.

Supplementary Figure 2

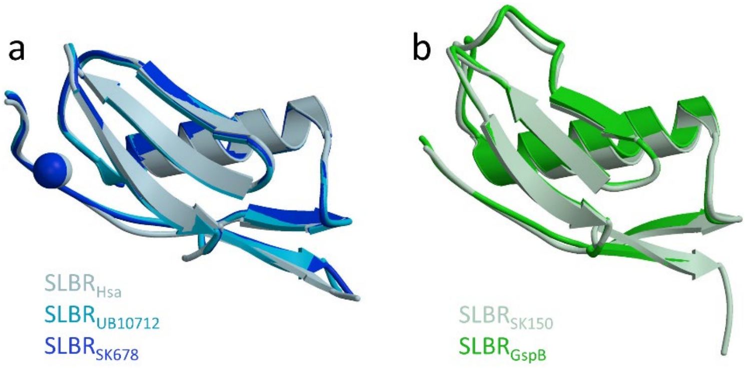

Supplementary Figure 2. Comparison of the Unique domain of bacterial SLBRs. Overlay of the Unique domains of: **a** $SLBR_{Hsa}$, $SLBR_{UB10712}$ and $SLBR_{SK678}$ and **b** $SLBR_{GspB}$ and $SLBR_{SK150}$. Ions are shown as spheres in the color corresponding to the SLBR. Based on the composition of the crystallization solution, the ions were tentatively assigned as Na^{+} in $SLBR_{Hsa}$, Ca^{2+} in $SLBR_{SK678}$ and Ca^{2+} in $SLBR_{UB10712}$. The view is rotated as compared to **Fig. 3** in order to highlight the structural similarity of this domain across the different branches of the phylogenetic tree.

Supplementary Figure 3

SLBR$_{Hsa}$-sTa

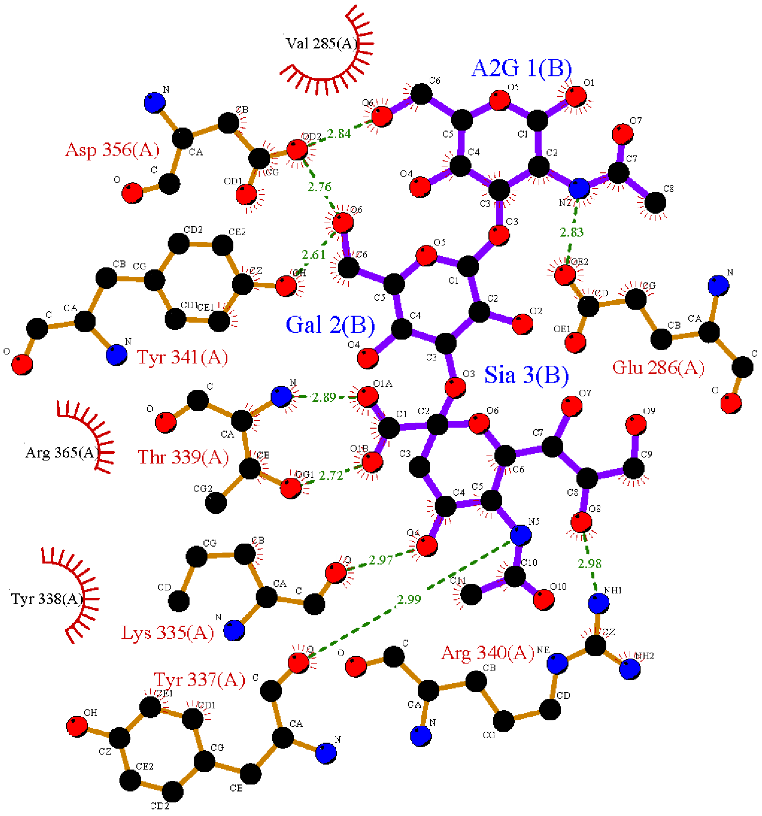

Supplementary Figure 3. Ligplot analysis of sTa bound to SLBR$_{Hsa}$. Residues interacting with the ligand via electrostatic interactions are indicated by a red halo. Hydrogen bonds are shown by a sticks and spheres representation of the amino acid and a dashed line connecting to the ligand. A2G is GalNAc, and Sia is Neu5Ac.

Supplementary Figure 4

SLBR$_{Hsa}$-sLec

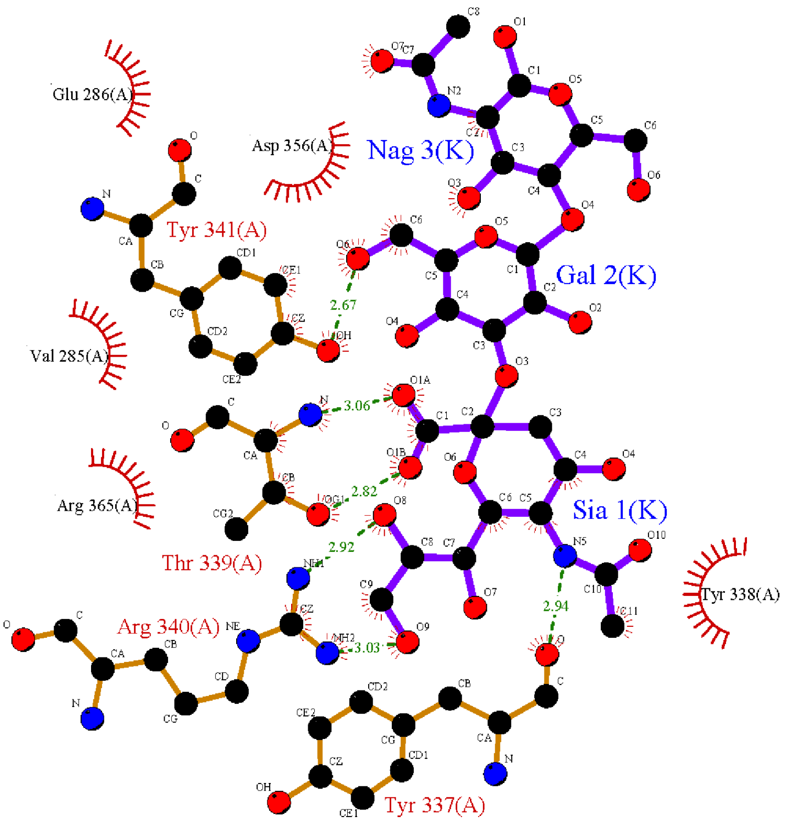

Supplementary Figure 4. Ligplot analysis of sLe^{C} bound to SLBR$_{Hsa}$. Residues interacting with the ligand via electrostatic interactions are indicated by a red halo. Hydrogen bonds are shown by a sticks and spheres representation of the amino acid and a dashed line connecting to the ligand. Nag is GlcNAc, and Sia is Neu5Ac.

Supplementary Figure 5

SLBR$_{Hsa}$-3sLn

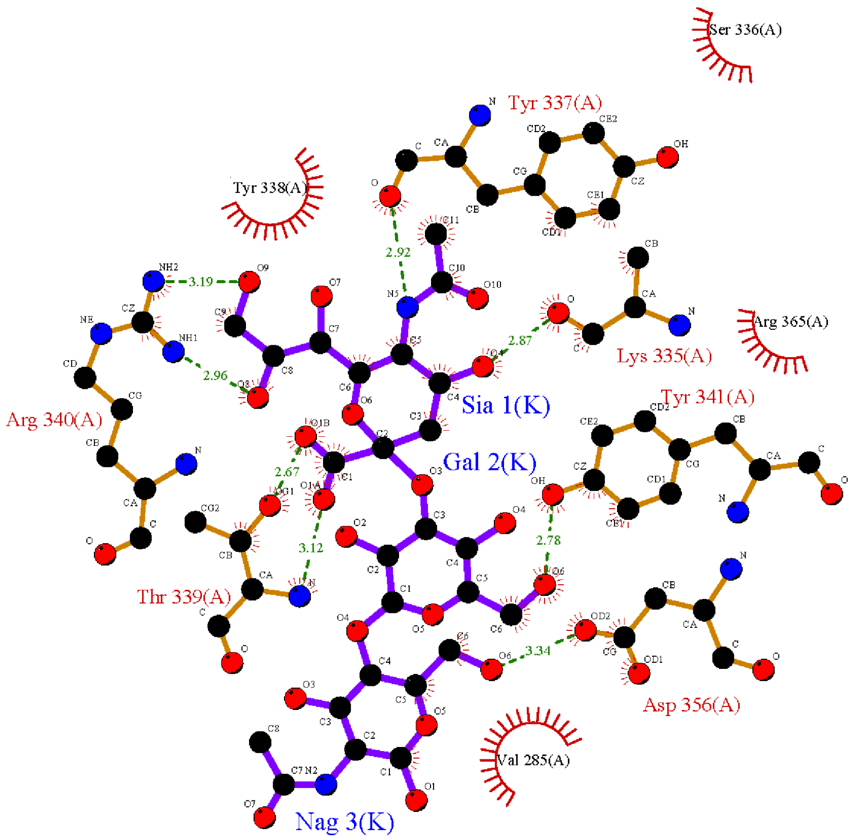

Supplementary Figure 5. Ligplot analysis of 3'sLn bound to SLBR$_{\mathbf{Hsa}}$. Residues interacting with the ligand via electrostatic interactions are indicated by a red halo. Hydrogen bonds are shown by a sticks and spheres representation of the amino acid and a dashed line connecting to the ligand. A2G is GalNAc, and Sia is Neu5Ac.

Supplementary Figure 6

SLBR$_{Hsa}$-6S-sLex

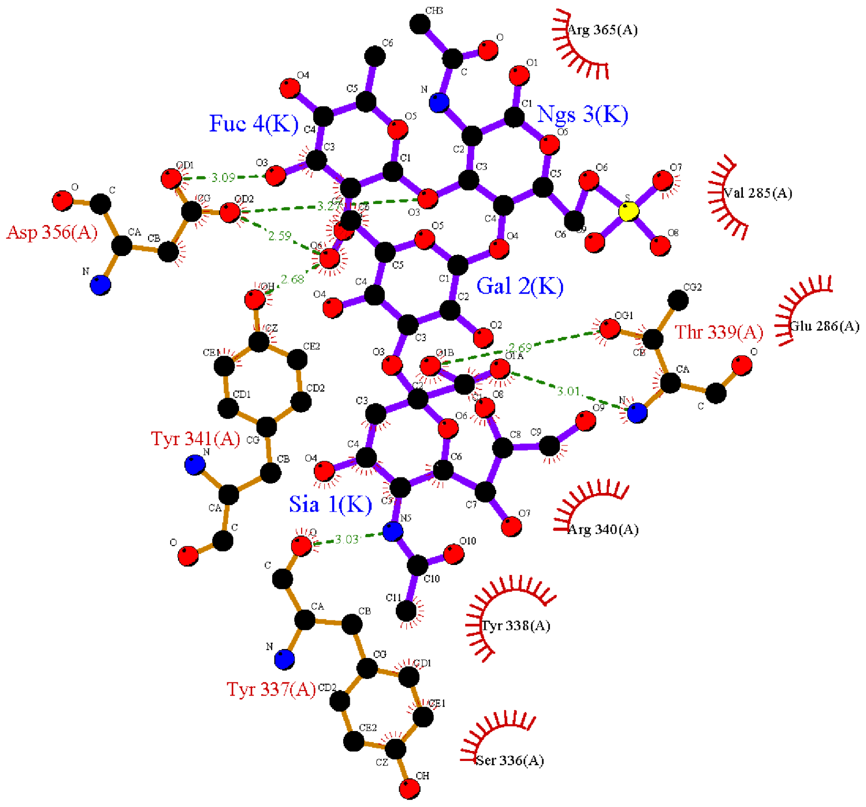

Supplementary Figure 6. Ligplot analysis of 6S-sLe^{X} bound to SLBR$_{Hsa}$. Residues interacting with the ligand via electrostatic interactions are indicated by a red halo. Hydrogen bonds are shown by a sticks and spheres representation of the amino acid and a dashed line connecting to the ligand. Ngs is GlcNAc, and Sia is Neu5Ac.

Supplementary Figure 7

SLBR$_{GspB}$-sTa

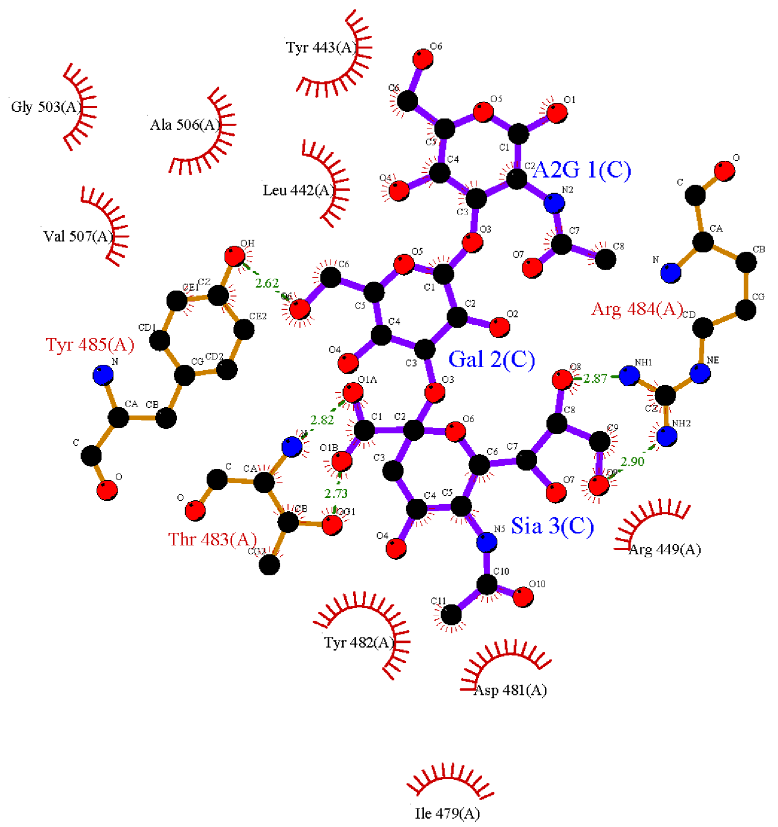

Supplementary Figure 7. Ligplot analysis of sTa bound to SLBR$_{GspB}$. Residues interacting with the ligand via electrostatic interactions are indicated by a red halo. Hydrogen bonds are shown by a sticks and spheres representation of the amino acid and a dashed line connecting to the ligand. A2G is GalNAc, and Sia is Neu5Ac.

Supplementary Figure 8

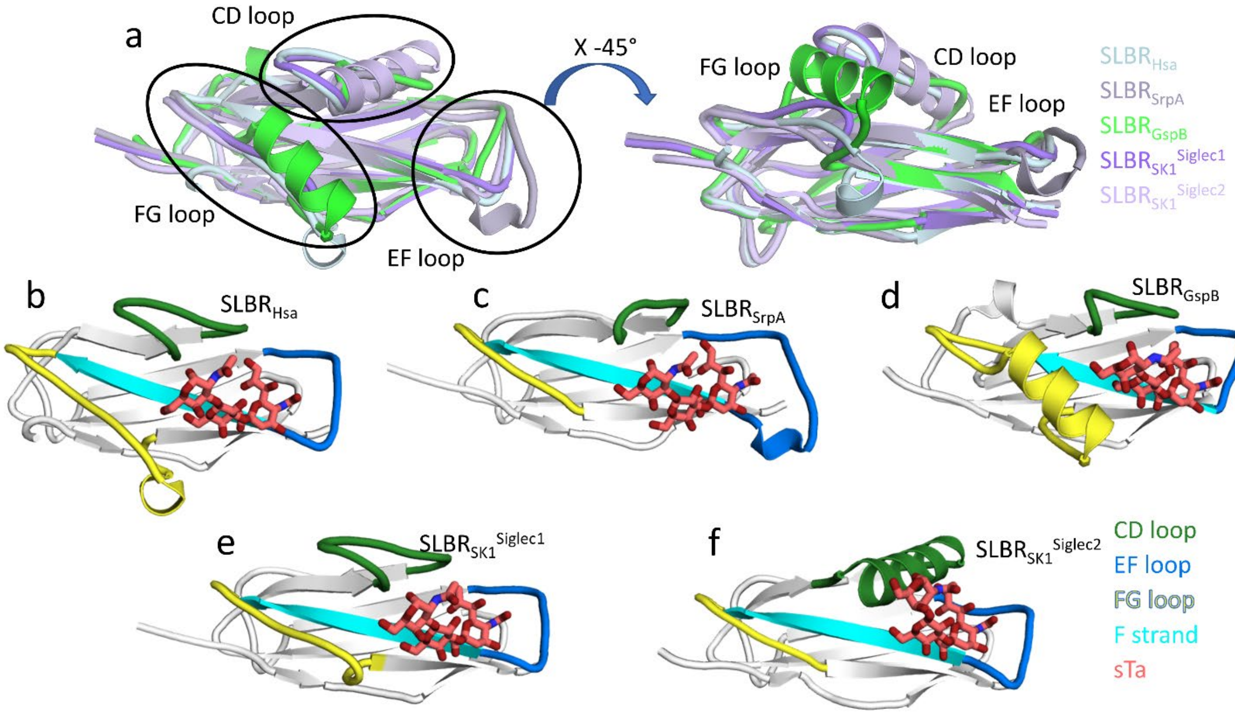

Supplementary Figure 8. Selectivity loops in sTa-bound SLBRs. Various SLBRs bound to sTa are shown in cartoon, and sTa is shown in *gray* sticks with oxygen colored *red* and nitrogen colored *blue*. **a** Overlay of the sTa-bound SLBRs shown in **b-f**. $SLBR_{Hsa}$, $SLBR_{SrpA}$[6], and $SLBR_{GspB}$[1] are shown in *blue-gray*, *gray*, and *green* respectively. **h-f** The $SLBR_{SK1(a)}$ and $SLBR_{SK1(b)}$[7] are shown in *purple* and *lavender* respectively.

Supplementary Figure 9

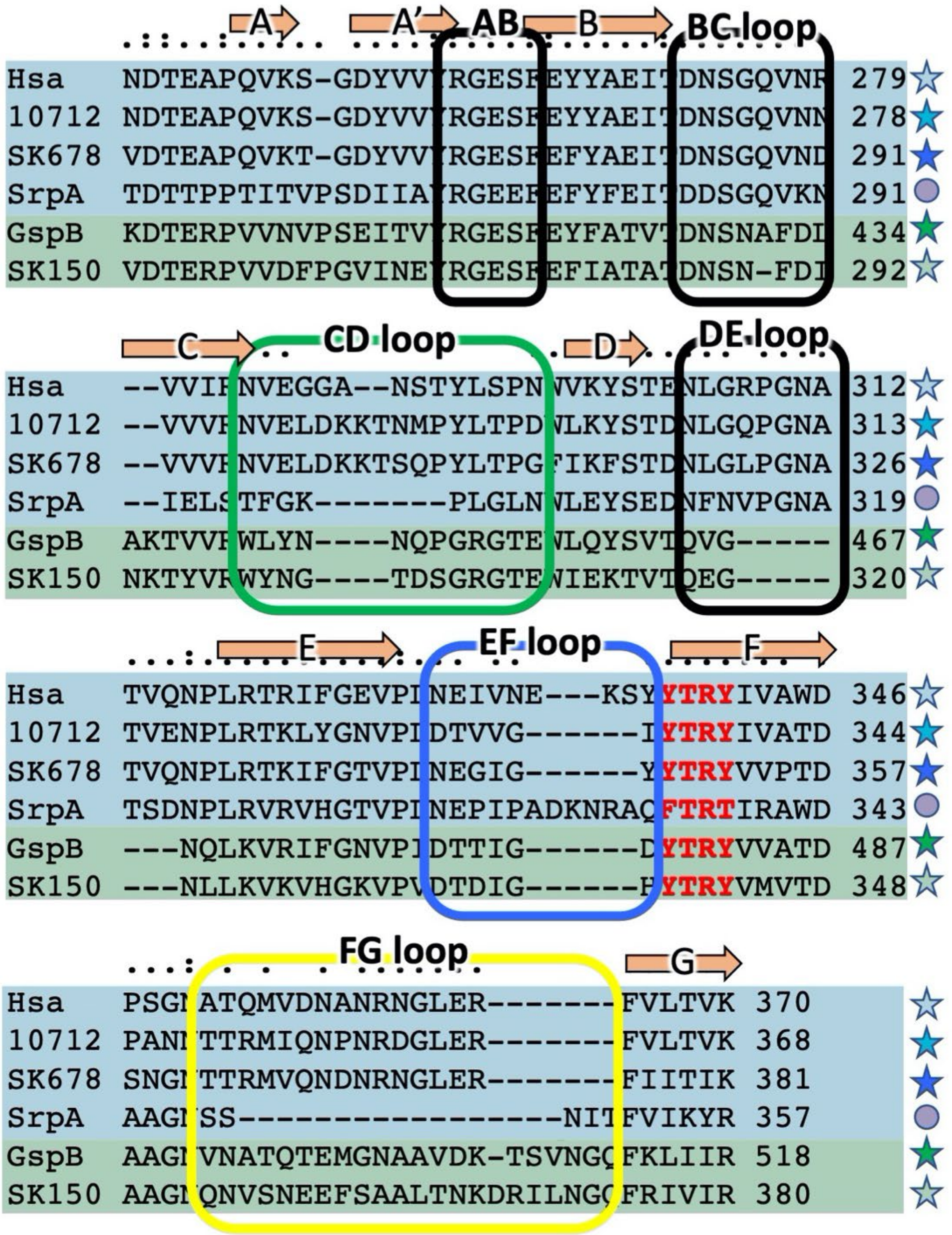

Supplementary Figure 9. Sequence alignment of the Siglec domain of select SLBRs. Sequences of the evolutionary-grouped SLBRs similar to $SLBR_{Hsa}$ are highlighted with a blue background. As comparators, sequences of $SLBR_{GspB}$ and an additional comparator in the same branch of the evolutionary tree, $SLBR_{SK150}$, are shown with a green background. Strands conserved in the V-set Ig fold are indicated, and residues of the interstrand loops are highlighted with boxes. The CD (*green*), EF (*blue*), and FG (*yellow*) loops disproportionately differ in length and homology. The ΦTRX motif on the F-strand of the Ig fold is highlighted with red text.

Supplementary Figure 10

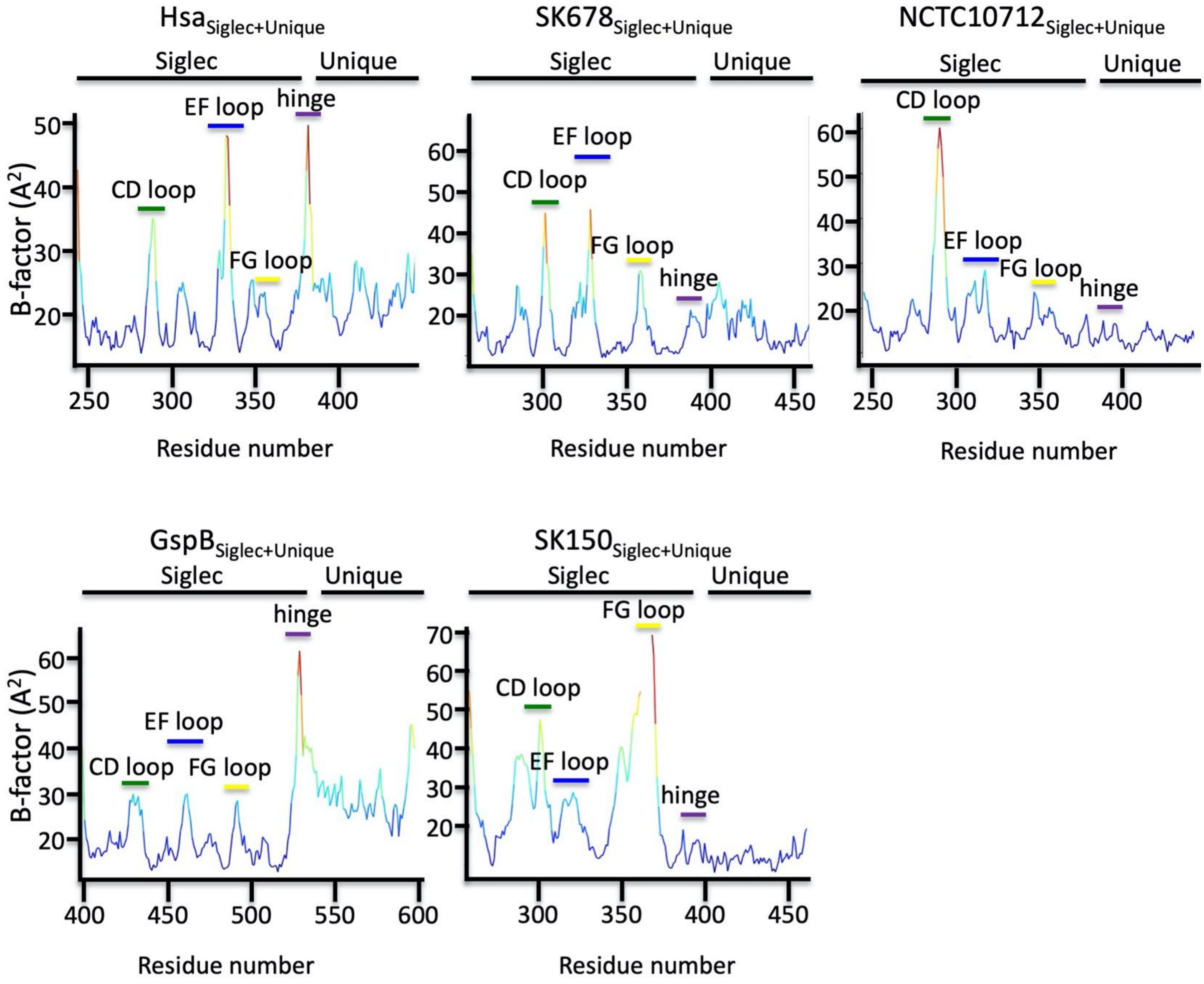

Supplementary Figure 10. Temperature factor analysis of unliganded SLBR structures. For each graph, the residue number is on the x-axis, and the crystallographic temperature factor (B-factor) is on the y-axis. Coloring is by relative B-factor. Regions with the lowest B-factors are predicted to have the lowest mobility (*dark blue*); regions with the highest B-factors are predicted to have the highest mobility (*red*).

Supplementary Figure 11

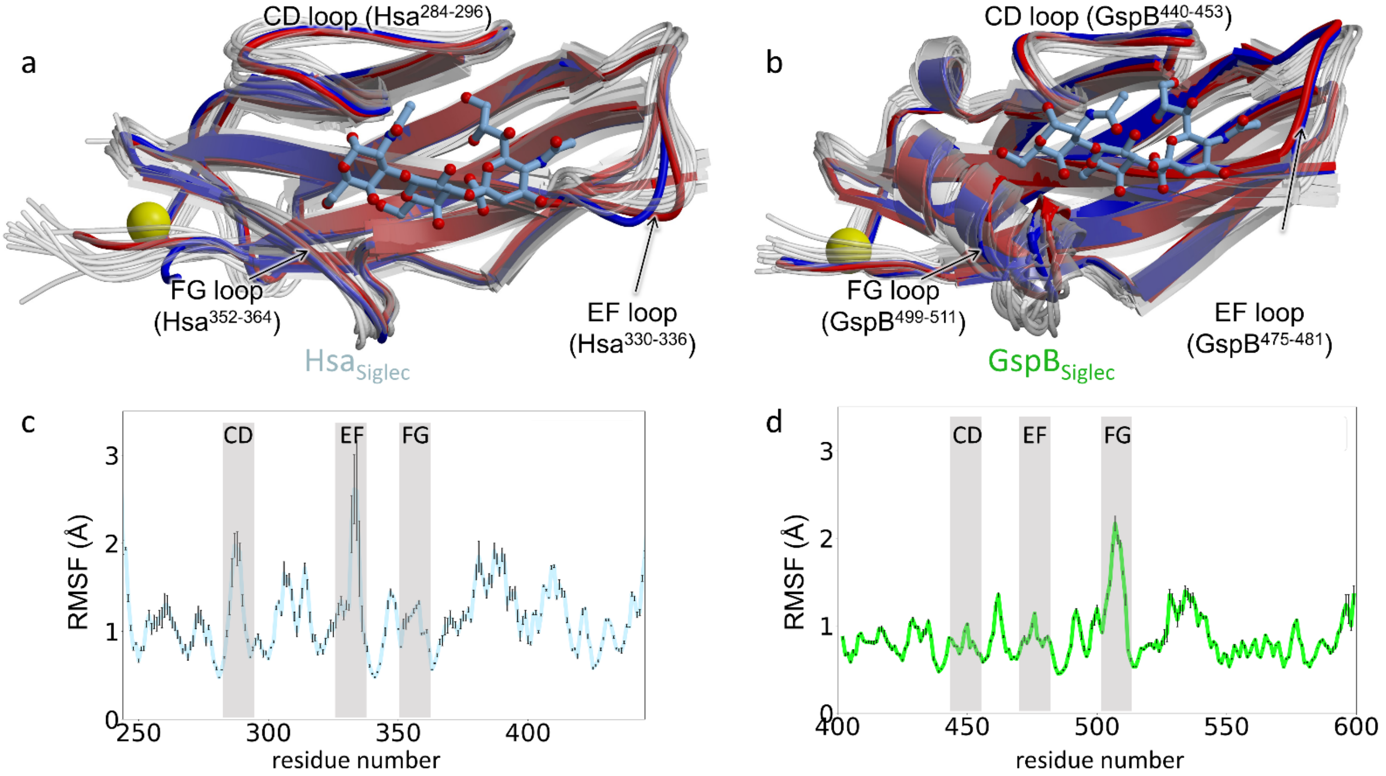

Supplementary Figure 11. MD simulations of SLBRs. a,b Superposition of a representative subset of MD simulation snapshots (*translucent*) of **a** The Siglec domain of $SLBR_{Hsa}$ and **b** $SLBR_{GspB}$ onto the crystal structures determined in the presence (*blue*) and absence (*red*) of the sTa sialoglycan. MD simulations used structures determined in the absence of ligand as a starting point. **c,d** Root mean square fluctuations (RMSF) of the Siglec domain of **c** $SLBR_{Hsa}$ and **d** $SLBR_{GspB}$ are plotted for each residue as compared to the average position of the Cα atoms of each residue. Calculations were performed on the adjacent Siglec and Unique domains, with only the resected Siglec domain shown. Error bars are shown in black lines at each residue and correspond to the standard error over 3 independent simulations. The positions of the CD, EF, and FG loops within the sequence are shown with a grey background. In $SLBR_{Hsa}$, the EF loop exhibits the largest predicted mobility of any region within the adhesin. In contrast, in $SLBR_{GspB}$, the FG loop exhibits the largest mobility.

Supplementary Figure 12

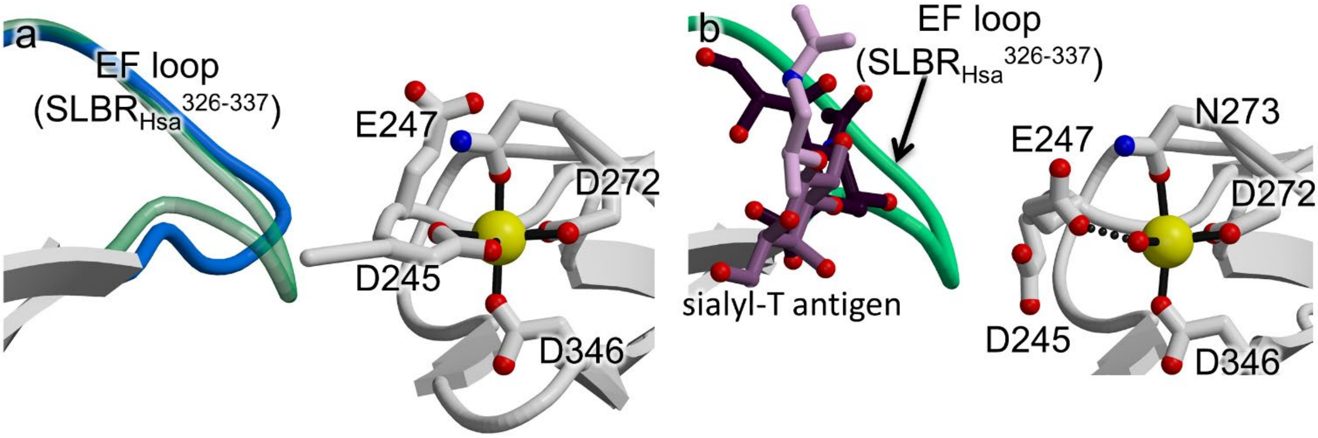

Supplementary Figure 12. Crystal packing and conformational change upon ligand binding in SLBR$_{Hsa}$. **a** Crystal contact between the EF loop of unliganded SLBR$_{Hsa}$ (*blue*) and the N-terminus of a neighboring molecule. The position of the loop in SLBR$_{Hsa}$ bound to sTa (*transparent green*) would be in steric conflict with the N-terminus of the adjacent molecule in the absence of a conformational change. **b** Change in crystal contact following binding to sTa. When the EF loop closes over sTa, the N-terminus undergoes a compensatory conformational adjustment that changes the coordination sphere of a labile cation in the neighboring molecule. Specifically, the main chain of SLBR$_{Hsa}^{D245}$ normally coordinates the ion but would be in steric conflict with ligand-bound position of the EF loop. Following the conformational change, SLBR$_{Hsa}^{E247}$ now coordinates the ion. This crystal contact likely creates an energy minimum and shifts the conformational equilibrium of the EF loop toward the open position, even in the presence of glycan. Adjustment of the EF loop to ligand is observed only in a subset of the costructures, but it is expected to close over ligand when in solution.

Supplementary Figure 13

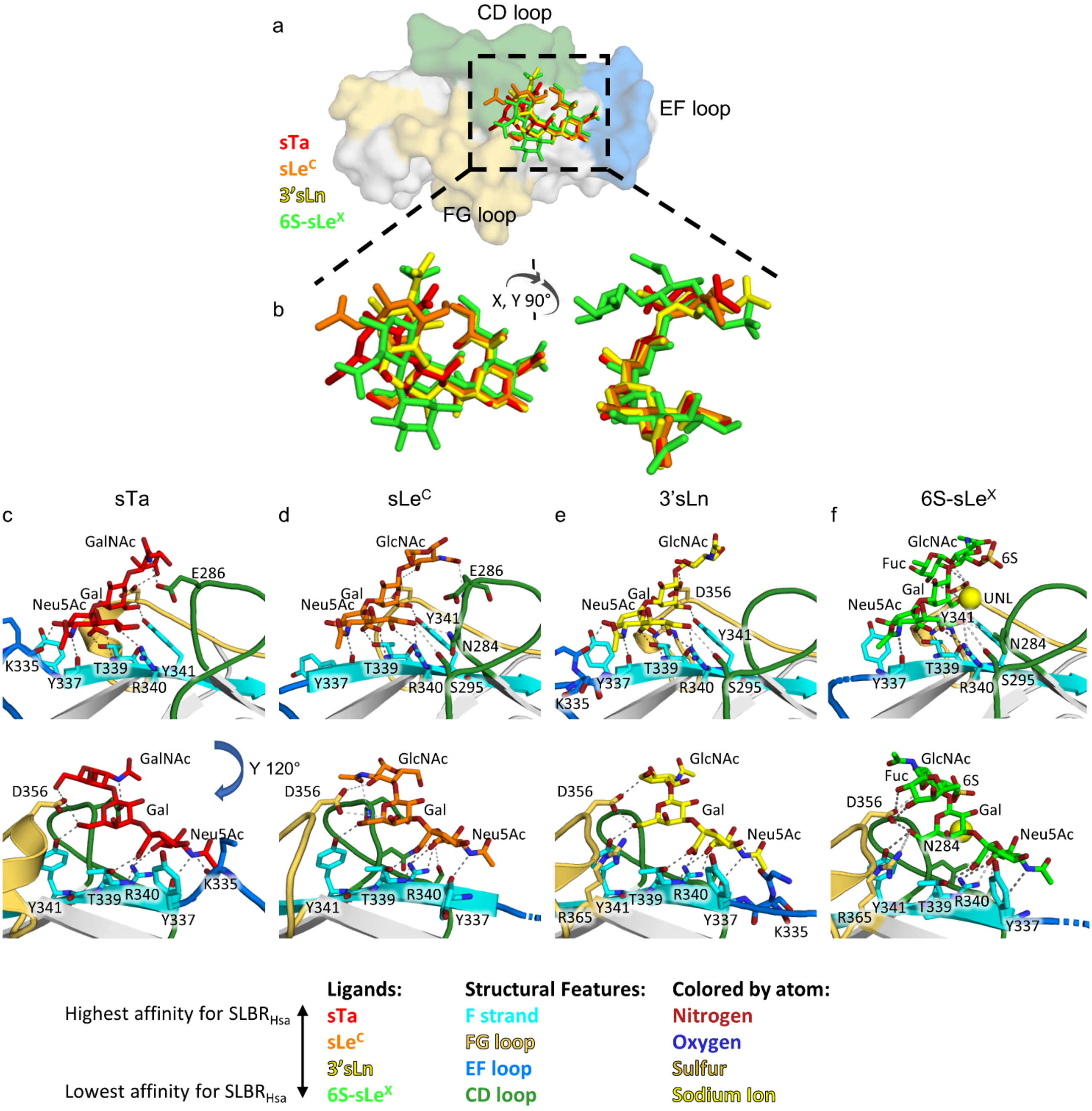

Supplementary Figure 13. Sialoglycan position in the SLBR$_{Hsa}$ binding pocket. a-f The sialoglycan ligands sTa, sLe^{C}, 3'sLn, and 6S-sLe^{X} are shown in *red*, *orange*, *yellow*, and *green*, where *red* is sTa, which is the highest affinity ligand for SLBR$_{Hsa}$, and *green* is 6S-sLe^{X} the lowest affinity ligand for SLBR$_{Hsa}$ used in this study. **a** sTa-bound SLBR$_{Hsa}$ is shown as a *grey* surface. **b** Close-up view of the overlaid sialoglycan ligands. The position of 6S-sLe^{X} is shifted by ~0.8Å from the position of the highest affinity ligand, sTa. **c-f** The binding pocket of SLBR$_{Hsa}$ is shown in cartoon with the CD loop colored *green*, the EF loop colored *blue*, and the FG loop colored *yellow*. The F strand containing the ϕTRX motif is shown in cyan. The unknown ligand (UNL) in the 6S-sLe^{X} structure is shown as a *yellow sphere*. The sialoglycan ligands and residues that participate in hydrogen bonding with the ligands are shown in sticks with nitrogen shown in *blue* and oxygen shown in *red*. Hydrogen bonds and electrostatic interactions between SLBR$_{Hsa}$ and the sialoglycans are shown as *dark grey* and *light grey* dashed lines respectively.

Supplementary Figure 14

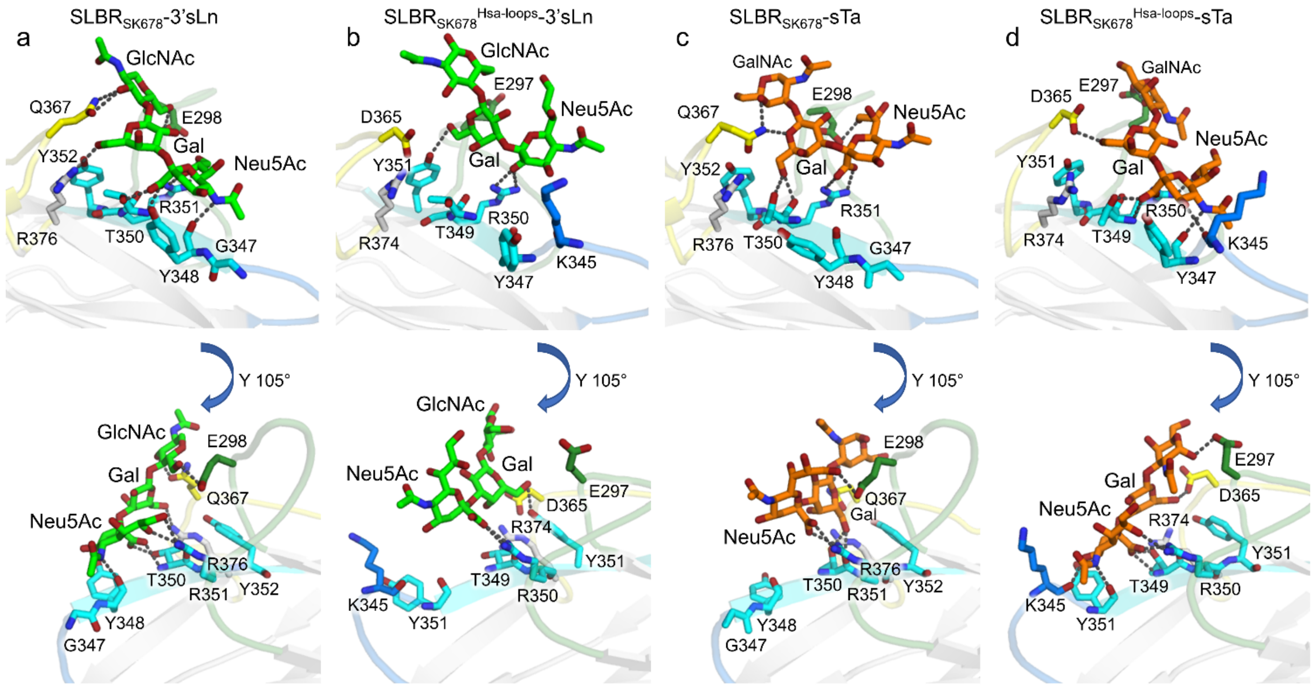

Supplementary Figure 14. Computational analysis of SLBR$_{SK678}^{Hsa\text{-}loops}$ binding to ligands. a-d computational structures of 3'sLn and sTa bound SLBR$_{SK678}$ and the SLBR$_{SK678}^{Hsa\text{-}loops}$ chimera. **a-b** The loops of the SLBR$_{SK678}^{Hsa\text{-}loops}$ chimera are predicted to make additional interactions between the F strand and sTa when compared to the parent SLBR$_{SK678}$. **c-d** The SLBR$_{SK678}^{Hsa\text{-}loops}$ chimera is predicted to lose interactions between the EF loop, CD loop, and the F strand; and 3'sLn when compared to parent SLBR$_{SK678}$. The lower affinity complexes, **a** sTa bound SLBR$_{SK678}$ and **d** 3'sLn bound SLBR$_{SK678}^{Hsa\text{-}loops}$ both lack interactions between the C-terminal end of the F strand and the sialic acid moiety.

Supplementary Figure 15

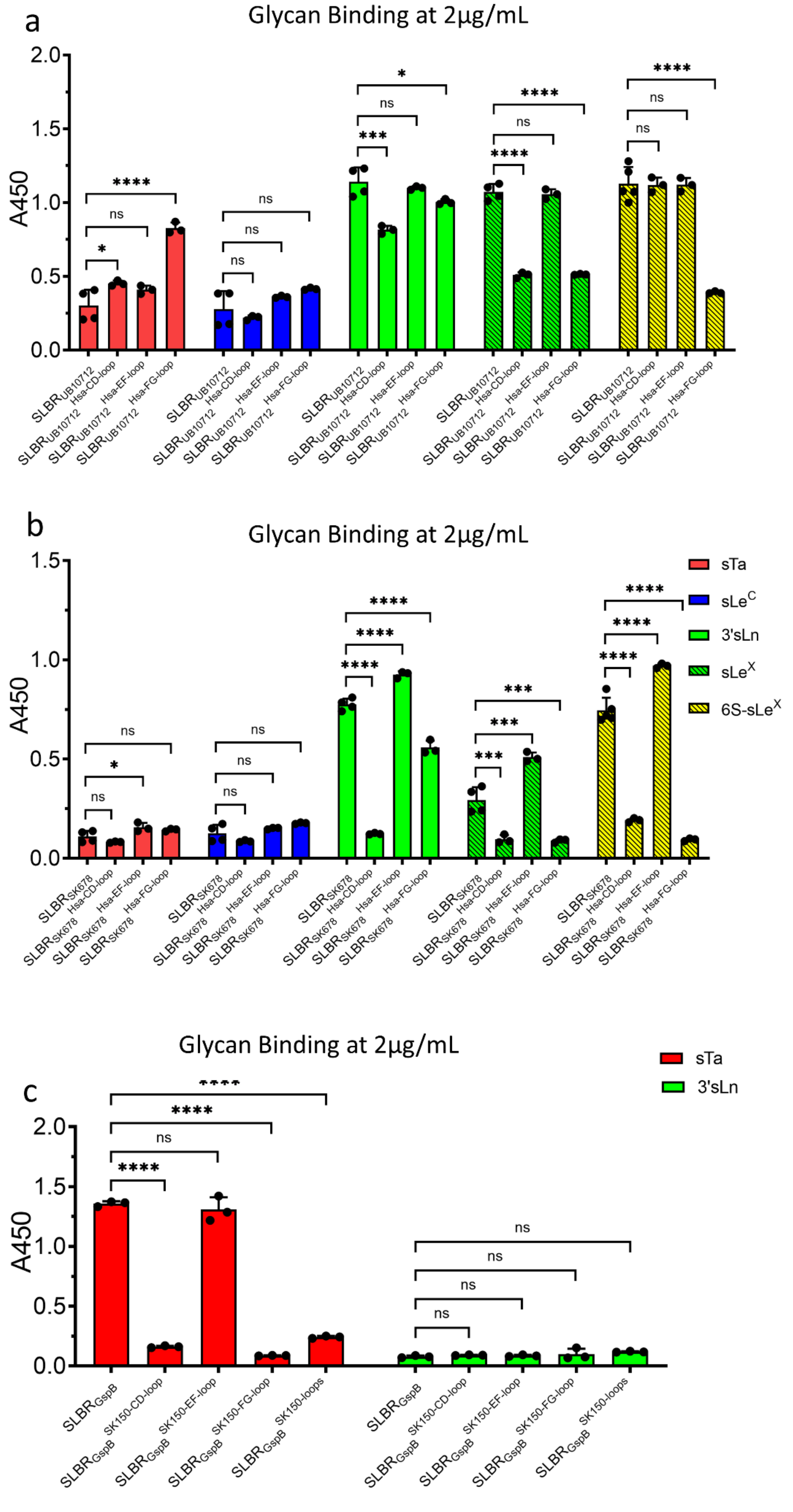

Supplementary Figure 15. Quantitation of glycan binding by the single-loop chimeras of SLBRs. Binding of biotin-glycans (2 μg/ml) to **a** GST-SLBR$_{UB10712}$ containing loops CD, EF, or FG of SLBR$_{Hsa}$, substituted individually; **b** GST-SLBR$_{SK678}$ containing loops CD, EF, or FG of SLBR$_{Hsa}$, substituted individually. **a** Loop substitution did not significantly affect binding to sLe^{C}. FG loop substitution led to a decrease in binding sulfate sLe^{X}. **b** SLBR$_{SK678}$$^{Hsa\text{-}CD\text{-}loop}$ exhibited substantially decreased affinity for 3'sLn

and 6S-sLe^X, SLBR$_{SK678}^{Hsa\text{-}EF\text{-}loop}$ had an increase in binding to all tested ligands except for sLe^C. SLBR$_{SK678}^{Hsa\text{-}FG\text{-}loop}$ had a moderately decreased affinity for 3'sLn, and a substantially decreased affinity for sLe^X and 6S-sLe^X. SLBR$_{UB10712}^{Hsa\text{-}CD\text{-}loop}$ exhibited a moderate loss of affinity to 3'sLn and sLe^X and a moderate gain of affinity to sTa; SLBR$_{UB10712}^{Hsa\text{-}FG\text{-}loop}$ exhibited a decrease in affinity to sLe^X and 6S-sLe^X and a moderate increase of affinity to sTa. **c** Binding of biotin-glycans (1 µg/ml) to GST-SLBR$_{GspB}$ containing loops CD, EF, or FG of SLBR$_{SK150}$ substituted either individually or together. With the exception of SLBR$_{GspB}^{SK150\text{-}EF\text{-}loop}$, all SLBR$_{GspB}$-SLBR$_{SK150}$ chimeras showed a substantial reduction in binding. Values correspond to the mean ± standard deviation, with n = 3 independent experiments using a single protein preparation. Binding of each glycan to each mutant was statistically compared to binding of the same glycan to the SLBR^{WT} with an ordinary one-way ANOVA corrected for multiple comparisons with Dunnett's test. Statistical significance is indicated by "*" (ns P >0.05; *P <0.05; ***P* < 0.01; ****P* < 0.001; **** < 0.0001)

Exact *p* values. In panel a, the *p* values for the comparison of binding of sTa to SLBR$_{UB10712}$ and variants SLBR$_{UB10712}^{Hsa\text{-}CD\text{-}loop}$, SLBR$_{UB10712}^{Hsa\text{-}EF\text{-}loop}$, and SLBR$_{UB10712}^{Hsa\text{-}FG\text{-}loop}$ are 0.0404, 0.155, and <0.0001 respectively. The *p* values for the comparison of binding of sLe^C to SLBR$_{UB10712}$ and variants SLBR$_{UB10712}^{Hsa\text{-}CD\text{-}loop}$, SLBR$_{UB10712}^{Hsa\text{-}EF\text{-}loop}$, and SLBR$_{UB10712}^{Hsa\text{-}FG\text{-}loop}$ are 0.599, 0.361, and 0.0811 respectively. The *p* values for the comparison of binding of 3'sLn to SLBR$_{UB10712}$ and variants SLBR$_{UB10712}^{Hsa\text{-}CD\text{-}loop}$, SLBR$_{UB10712}^{Hsa\text{-}EF\text{-}loop}$, and SLBR$_{UB10712}^{Hsa\text{-}FG\text{-}loop}$ are 0.0001, 0.703, and 0.350 respectively. The *p* values for the comparison of binding of sLe^X to SLBR$_{UB10712}$ and variants SLBR$_{UB10712}^{Hsa\text{-}CD\text{-}loop}$, SLBR$_{UB10712}^{Hsa\text{-}EF\text{-}loop}$, and SLBR$_{UB10712}^{Hsa\text{-}FG\text{-}loop}$ are <0.0001, 0.924, and <0.0001 respectively. The *p* values for the comparison of binding of 6S-sLe^X to SLBR$_{UB10712}$ and variants SLBR$_{UB10712}^{Hsa\text{-}CD\text{-}loop}$, SLBR$_{UB10712}^{Hsa\text{-}EF\text{-}loop}$, and SLBR$_{UB10712}^{Hsa\text{-}FG\text{-}loop}$ are 0.998, 0.999, and <0.0001 respectively. In panel **b** The *p* values for the comparison of binding of sTa to SLBR$_{SK678}$ and variants SK678$^{Hsa\text{-}CD\text{-}loop}$, SK678$^{Hsa\text{-}EF\text{-}loop}$, and SK678$^{Hsa\text{-}FG\text{-}loop}$ are 0.226, 0.0351, and 0.131 respectively. The *p* values for the comparison of binding of sLe^C to SLBR$_{SK678}$ and variants SLBR$_{SK678}^{Hsa\text{-}CD\text{-}loop}$, SLBR$_{SK678}^{Hsa\text{-}EF\text{-}loop}$, and SLBR$_{SK678}^{Hsa\text{-}FG\text{-}loop}$ are 0.158, 0.471, and 0.0626. The *p* values for the comparison of binding of 3'sLn to SLBR$_{SK678}$ and variants SLBR$_{SK678}^{Hsa\text{-}CD\text{-}loop}$, SLBR$_{SK678}^{Hsa\text{-}EF\text{-}loop}$, and SLBR$_{SK678}^{Hsa\text{-}FG\text{-}loop}$ are all <0.0001. The *p* values for the comparison of binding of sLe^X to SLBR$_{SK678}$ and variants SLBR$_{SK678}^{Hsa\text{-}CD\text{-}loop}$, SLBR$_{SK678}^{Hsa\text{-}EF\text{-}loop}$, and SLBR$_{SK678}^{Hsa\text{-}FG\text{-}loop}$ are 0.0004, 0.0002, and 0.0003 respectively. The *p* values for the comparison of binding of 6S-sLe^X to SLBR$_{SK678}$ and variants SLBR$_{SK678}^{Hsa\text{-}CD\text{-}loop}$, SLBR$_{SK678}^{Hsa\text{-}EF\text{-}loop}$, and SLBR$_{SK678}^{Hsa\text{-}FG\text{-}loop}$ are all <0.0001. In panel c, *p* values comparing binding of sTa to the parent SLBR$_{GspB}$ and the variants SLBR$_{GspB}^{SK150\text{-}CD\text{-}loop}$, SLBR$_{GspB}^{SK150\text{-}EF\text{-}loop}$, SLBR$_{GspB}^{SK150\text{-}FG\text{-}loop}$, and SLBR$_{GspB}^{SK150\text{-}loops}$ are <0.0001, 0.555, <0.0001, and <0.0001. The *p* values comparing binding of 3'sLn to the parent SLBR$_{GspB}$ and the variants SLBR$_{GspB}^{SK150\text{-}CD\text{-}loop}$, SLBR$_{GspB}^{SK150\text{-}EF\text{-}loop}$, SLBR$_{GspB}^{SK150\text{-}FG\text{-}loop}$, and SLBR$_{GspB}^{SK150\text{-}loops}$ are 0.881, 0.973, 0.623, and 0.122 respectively. Source data are provided as a Source Data file.

Supplementary Figure 16

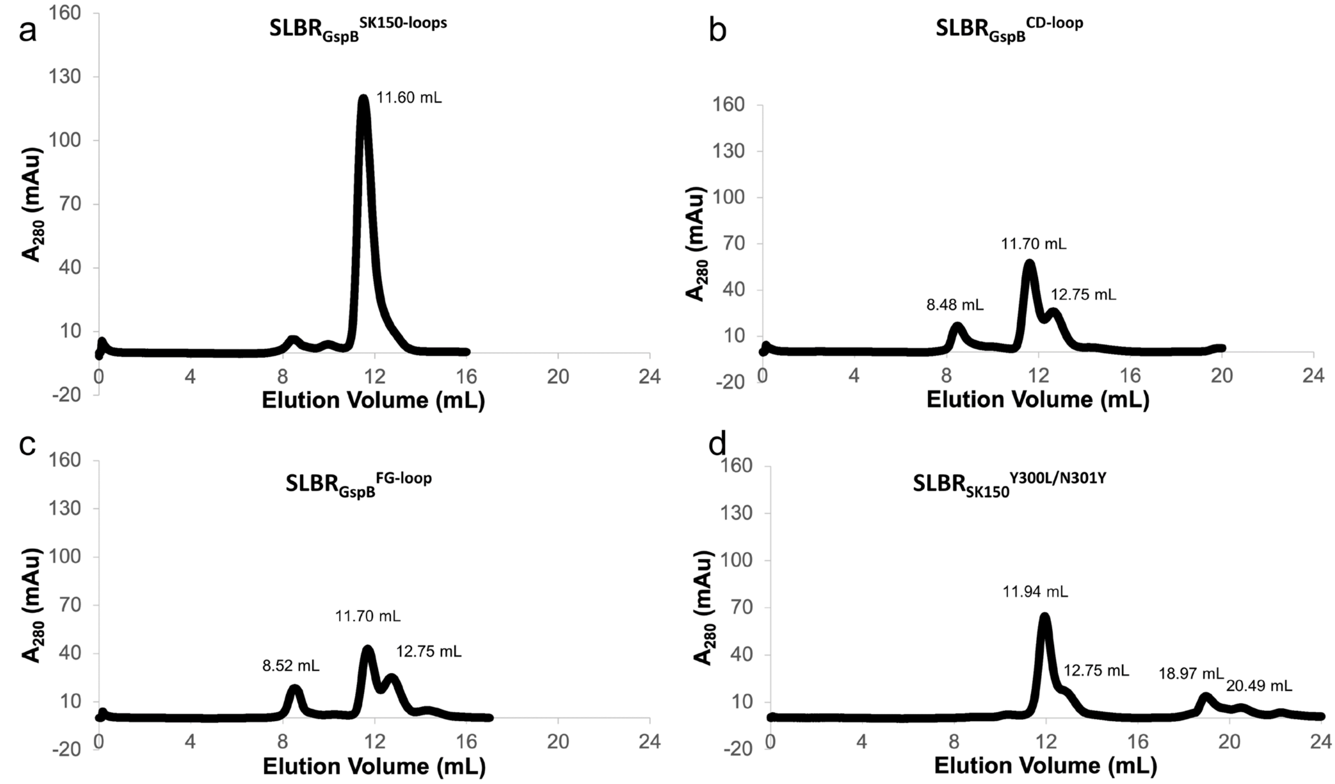

Supplementary Figure 16. Evaluation of protein folding in non-binding variant SLBR$_{GspB}$ and SLBR$_{SK150}$. For the GST-SLBRs that exhibited no binding to any tested glycan, folding was evaluated by observing the properties of the 0.5 mg non-binding variant SLBR on a 24-mL Superdex S200 size exclusion chromatography column. **a** GST-SLBR$_{GspB}$$^{SK150-loops}$ appears to be monodisperse and contains a single Gaussian peak with an elution volume of 11.60 mL. This profile is consistent with folded protein. **b** GST-SLBR$_{GspB}$$^{SK150-CD-loops}$ is a non-binding variant that exhibits loss of the peak corresponding to the folded protein, the appearance of an aggregate peak at 8.5 mL, and the appearance of a breakdown product at 12.75 mL. This is consistent with a deficit in either folding or stability. **c** SLBR$_{GspB}$$^{SK150-FG-loop}$ similarly exhibits a loss in the 11.6 mL peak and the appearance of both an aggregate peak and breakdown products. **d** The profile of SLBR$_{SK150}$$^{Y300L/N301Y}$ exhibits loss of the folded peak and appearance of breakdown products, but does not contain aggregates. **b-d** Decreases in sialoglycan binding of these variants as compared to wildtype (see Supplementary Figure 15C and Supplementary Figure 17F) may be due to the misfolding and stability loss observed here.

Supplementary Figure 17

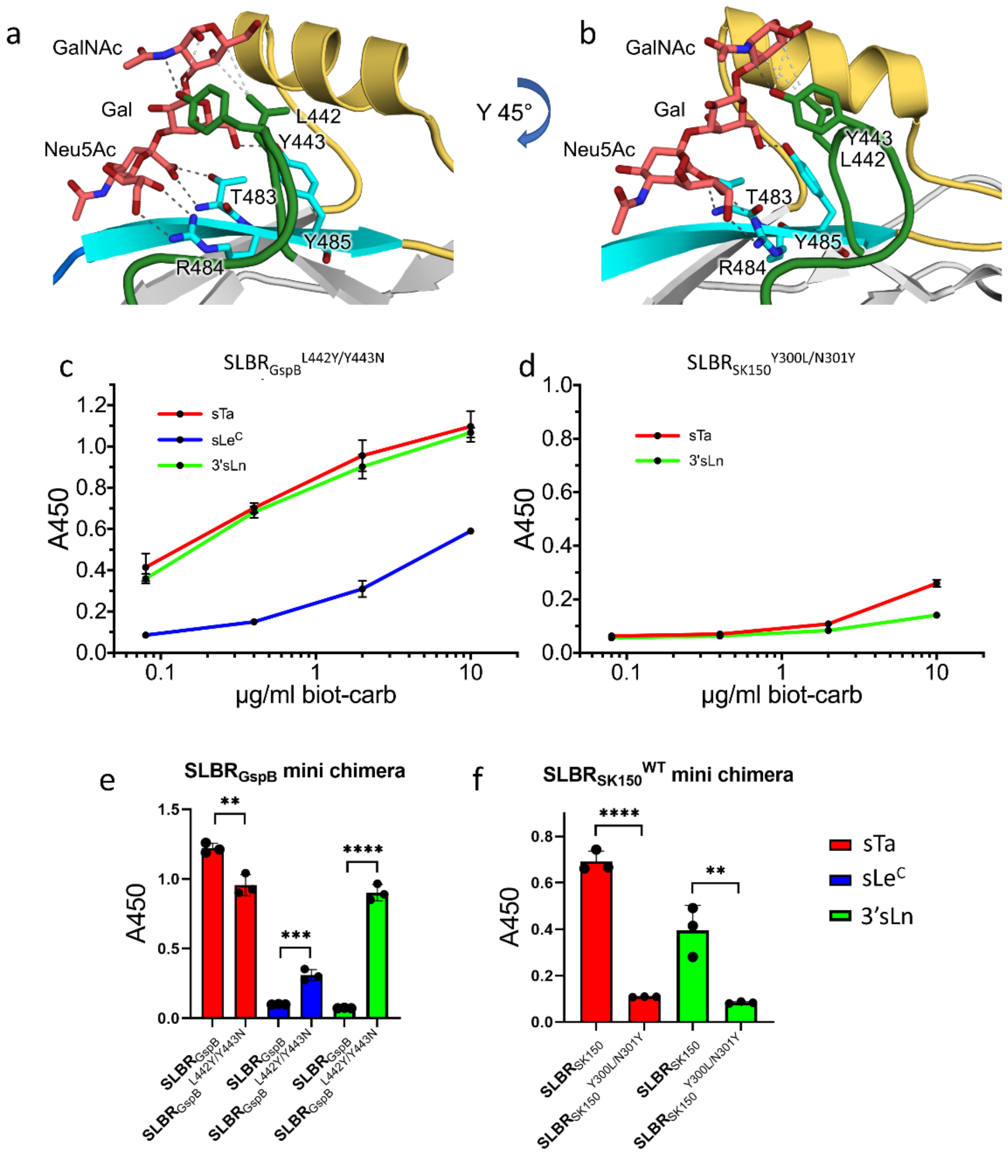

Supplementary Figure 17. Mini-chimeragenesis of SLBR$_{GspB}$ and SLBR$_{SK150}$. a-b Design of mini-chimeras was based upon examining the interactions between SLBR$_{GspB}$ and sTa. The F strand is colored *cyan*. The CD and FG loops are colored *green* and *yellow* respectively. sTa is shown in *salmon* with oxygens shown in *red* and nitrogens shown in *blue*. Hydrogen bonds are shown in *dark grey* dashed lines and electrostatic interactions are shown in *light grey* dashed lines. **c-d** Dose-response curves of biotin-glycan binding to immobilized binding regions (500 nM). Mean values are shown as black dots with the standard deviations represented by black bars. **c** Mini-chimeragenesis with SLBR$_{SK150}$ was accomplished with the GST-SLBR$_{GspB}^{L442Y/Y443N}$ double mutant. The mini-chimera becomes more broadly selective by increasing the affinity for 3'sLn and sLe^{C}. As a result, it exhibits binding selectivity more similar to wild-type GST-SLBR$_{SK150}$ (see Supplementary Figure 1). **d** The converse mini-chimeragenesis of SLBR$_{SK150}$ exhibited reduced binding for sialoglycan ligands that bind most avidly to both wild-type SLBR$_{GspB}$ and SLBR$_{SK150}$. **e-f** Comparison of parent and mini-chimera SLBR$_{GspB}$ and SLBR$_{SK150}$. Black circles represent individual data points and bars represent the mean ± SD. Binding of each glycan to the mini-chimera was compared to the parent SLBR using two-tailed parametric t tests. **e** The p values comparing binding of sTa, sLeC, and 3'sLn to SLBR$_{GspB}$ and SLBR$_{GspB}^{L442Y/Y443N}$ are 0.0054, 0.0008, and <0.0001 respectively. **f** The p values comparing binding of sTa and 3'sLn to SLBR$_{SK150}$ and SLBR$_{SK150}^{Y300L/N301Y}$ are <0.0001 and 0.0072 respectively. **c-f** n = 3 independent experiments using a single protein preparation. Statistical significance is indicated by ns: p

>0.05; *, p <0.05; **, $p < 0.01$; ***, $p < 0.001$; ****, $p < 0.0001$). Source data are provided as a Source Data file.

Supplementary Figure 18

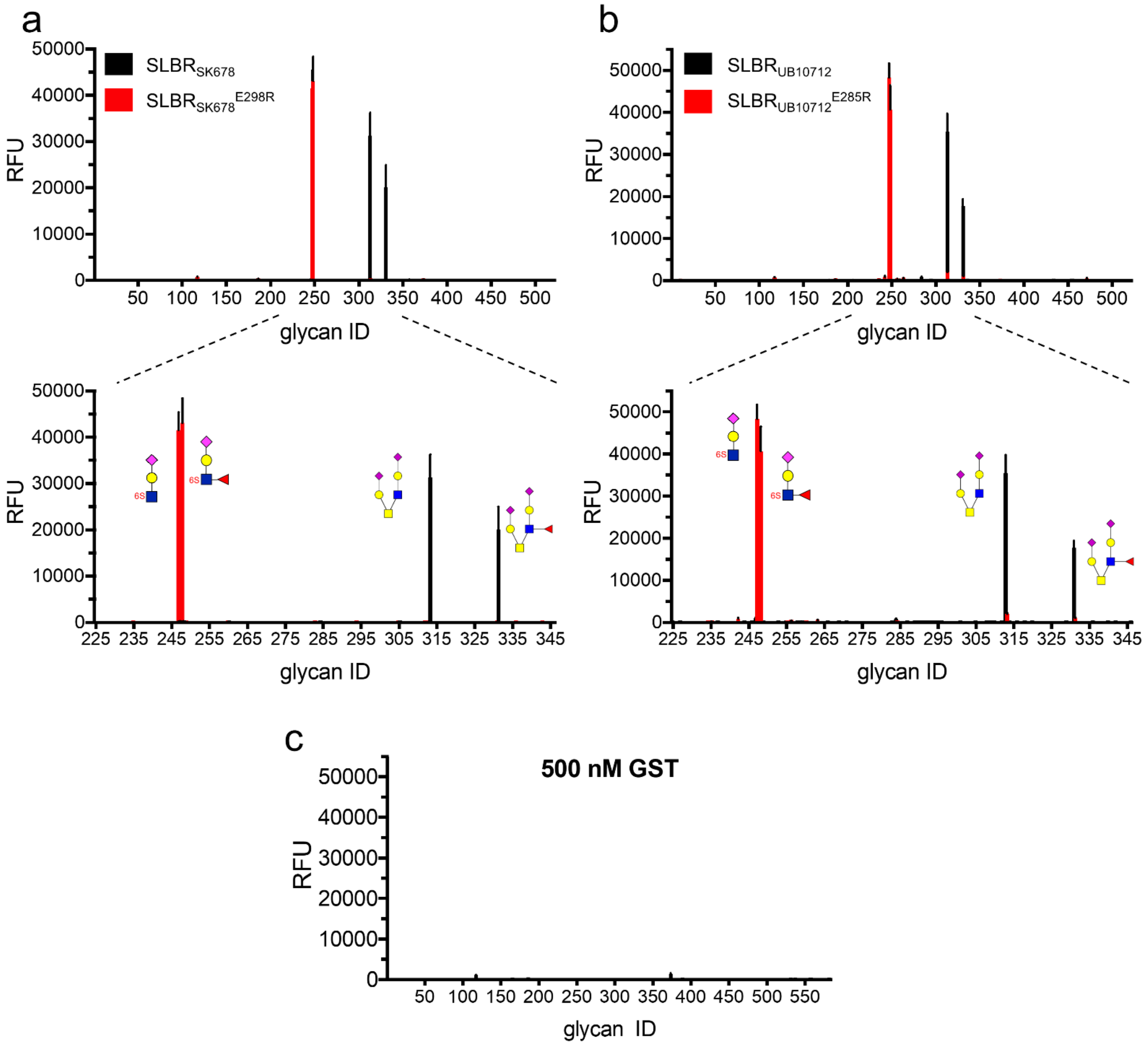

Supplementary Figure 18. Center for Functional Glycomics (CFG) version 5.4 glycan arrays for SLBR$_{SK678}$ and SLBR$_{SK678}^{E298R}$. Binding to 500 glycans in the CFG array was independently assessed to **a** GST-SLBR$_{SK678}$ (500 nM, *black*) and GST-SLBR$_{SK678}^{E298R}$ (500 nM, *red*); **b** GST-SLBR$_{UB10712}$ (500 nM, *black*) and GST-SLBR$_{UB10712}^{E285R}$ (500 nM, *red*); and **c** GST. The inset in **a** and **b** highlights the narrow selectivity and the difference in glycans that are robustly recognized by parent versus the engineered SLBRs. Numbers on the X-axis correspond to individual glycans in the arrays. The Y-axis is relative response.

Supplementary Figure 19

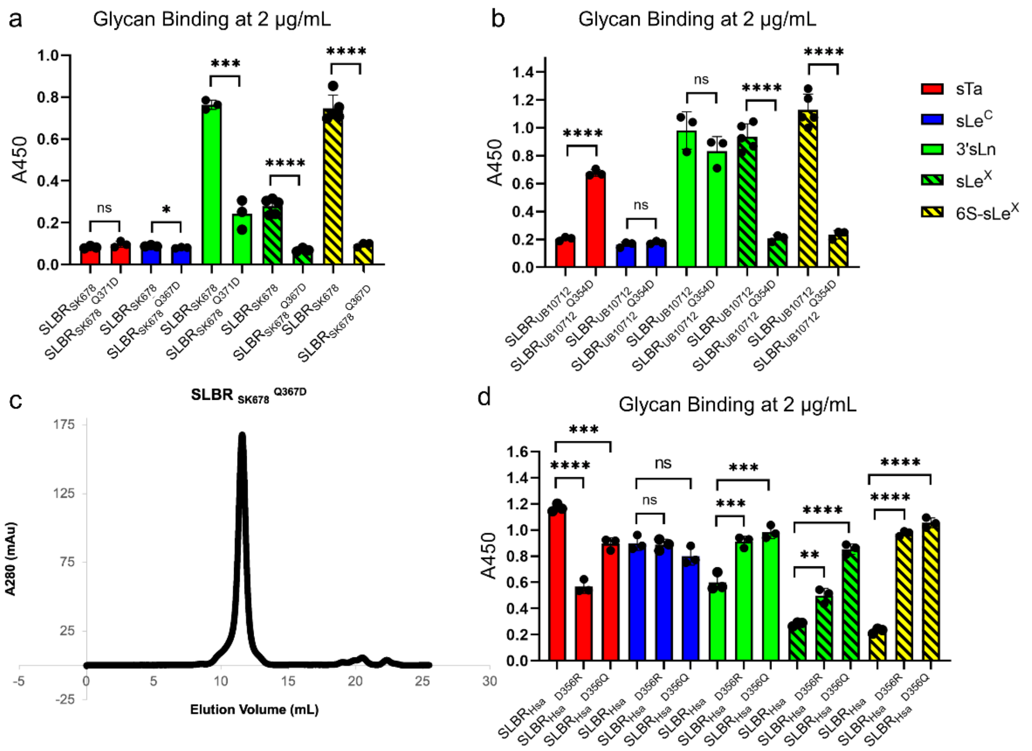

Supplementary Figure 19. Analysis of glycan binding by SLBRs with point mutations in the FG loop. A-B. Binding of glycans to each engineered variant SLBR was statistically compared to binding of the same glycan to parent SLBR using a two tailed nonparametric t test. **a** The *p* values comparing binding of sTa, sLe^{C}, 3'sLn, sLe^{X}, and 6S-sLe^{X} to $SLBR_{SK678}$ and $SLBR_{SK678}^{Q367D}$ variant are 0.201, 0.0456, 0.0003, <0.0001, and <0.0001 respectively. **b** The *p* values comparing binding of sTa, sLe^{C}, 3'sLn, sLe^{X}, and 6S-sLe^{X} to the parent $SLBR_{UB10712}$ to the variant $SLBR_{UB10712}^{Q354D}$ are <0.0001, 0.326, 0.205, <0.0001, and <0.0001 respectively. **c** Size exclusion chromatography of 0.5 mg of the non-binding $SLBR_{SK678}^{Q367D}$ variant shows a single peak at 11.6 mL, consistent with folded protein. **d** Binding of each glycan to $SLBR_{Hsa}$ FG mutants was statistically compared to binding of the same glycan to parent $SLBR_{Hsa}$ with an ordinary one-way ANOVA corrected for multiple comparisons with Dunnett's test. The *p* values comparing binding of sTa, sLe^{C}, 3'sLn, sLe^{X}, and 6S-sLe^{X} to the parent $SLBR_{Hsa}$ and the variant $SLBR_{Hsa}^{D356R}$ are <0.0001, 0.948, 0.0008, 0.0011, and <0.0001 respectively. The *p* values comparing binding of sTa, sLe^{C}, 3'sLn, sLe^{X}, and 6S-sLe^{X} to the parent $SLBR_{Hsa}$ and the variant $SLBR_{Hsa}^{D356Q}$ are 0.0004, 0.129, 0.0003, <0.0001, and <0.0001 respectively. **a,b,d** Black circles represent individual data points and bars represent the mean ± SD with n = 3 independent experiments using a single protein preparation. Statistical significance in each panel is indicated by: ns, $p > 0.05$; *, $p < 0.05$; **, $p < 0.01$; ***, $p < 0.001$; ****, $p < 0.0001$). Source data are provided as a Source Data file.

Supplementary Figure 20

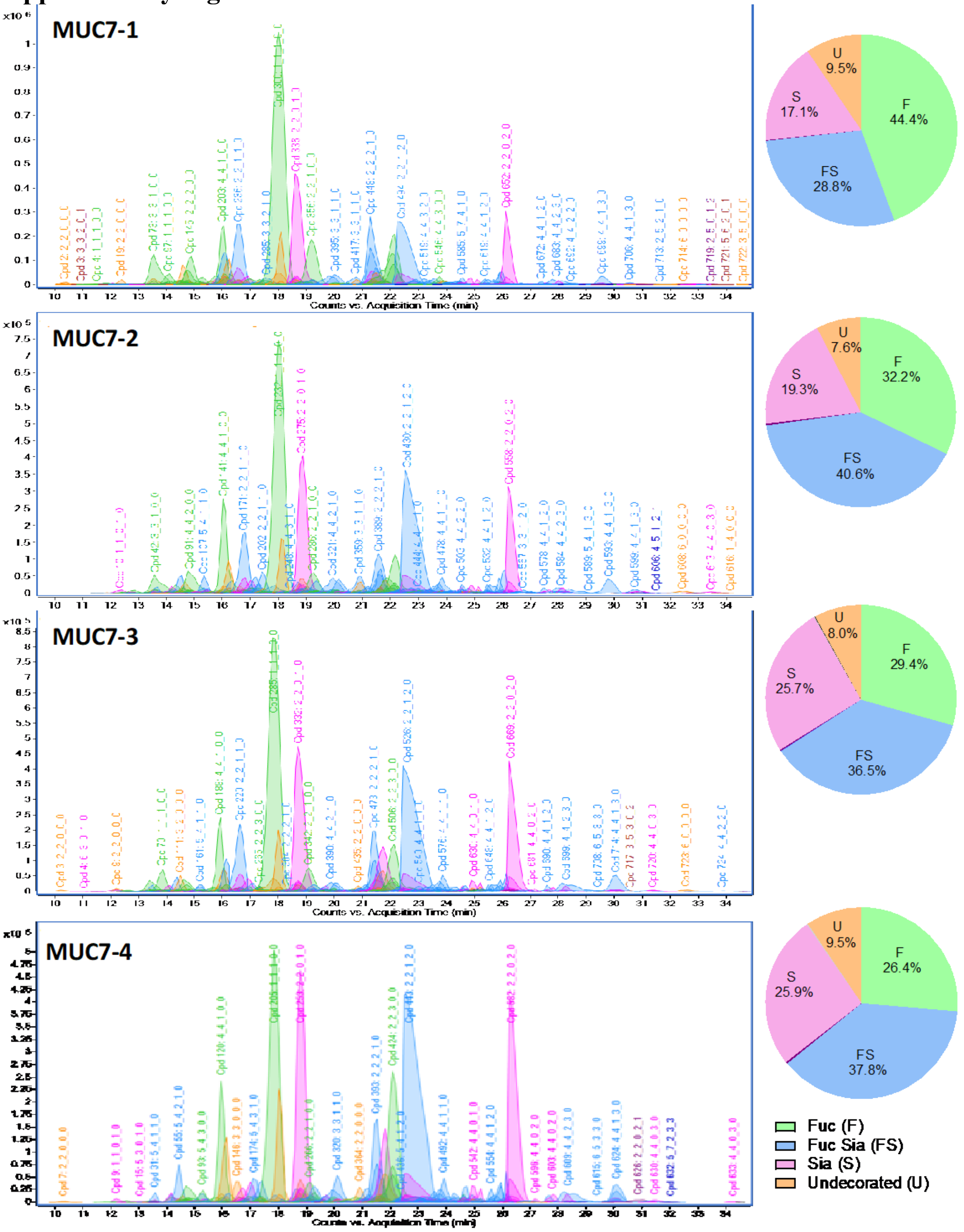

Supplementary Figure 20. Comparison of the identity of *O*-glycans released from four MUC7 samples, and pie charts representing the relative abundances of sub-glycan groups. The extracted compound chromatograms (ECCs) of *O*-glycans from four different saliva donors were categorized into four different groups: undecorated (U); fucosylated (F); sialylated (S); and fucosialylated (FS). The monosaccharide compositions (hexose (Hex)-HexNAc-Fuc-NeuAc-Sulf) were inferred from the precise masses determined by LC-MS. Source data are provided as a Source Data file.

Supplementary Figure 21

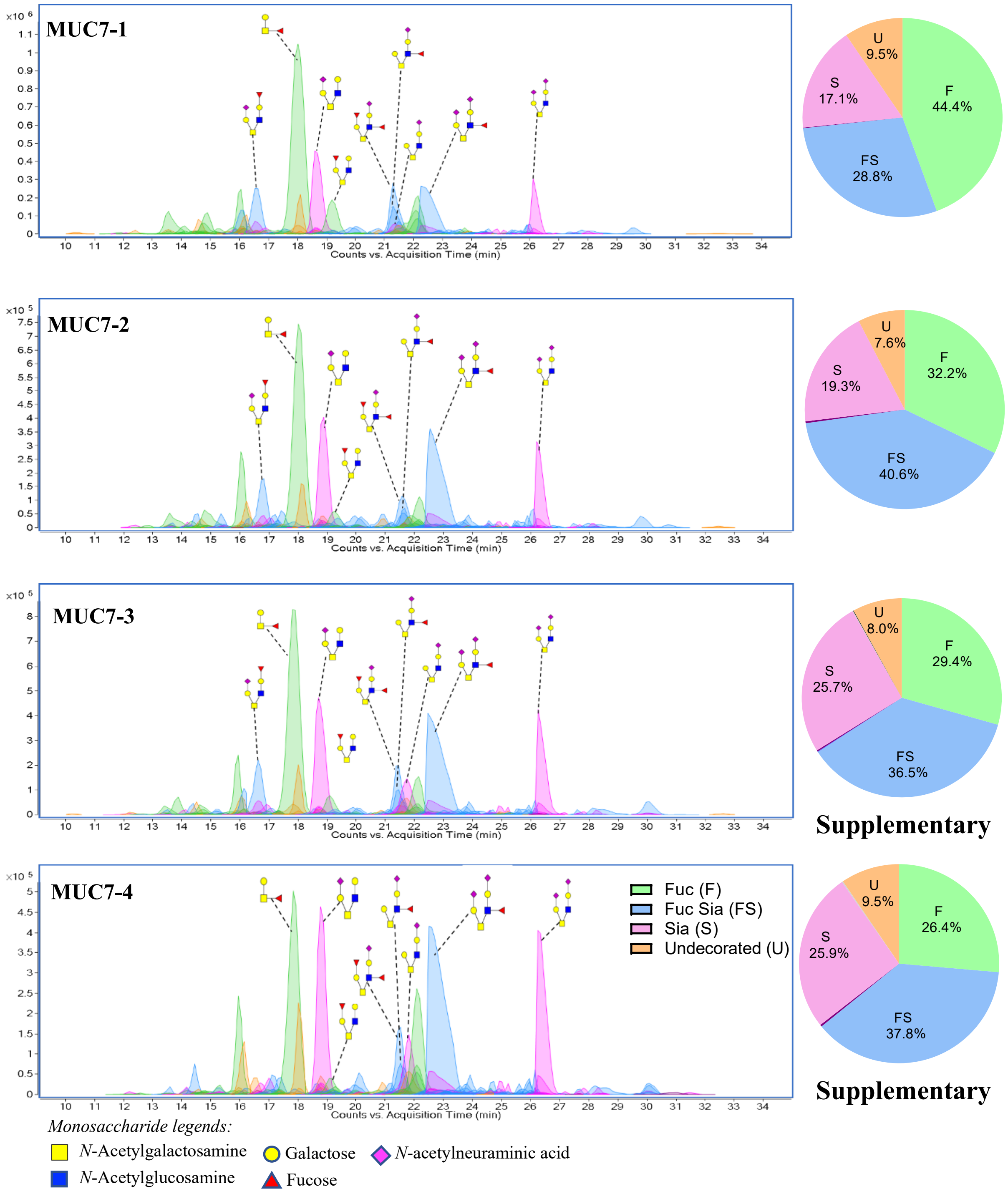

Supplementary Figure 21.

Supplementary Figure 21.

Supplementary Figure 21. Proposed structures of the major *O*-glycans of MUC7 from four donors. The putative structures are based on the precise masses and inferred monosaccharide compositions in addition to the MS/MS fragmentation patterns and literature data. Source data are provided as a Source Data file.

Supplementary Figure 22

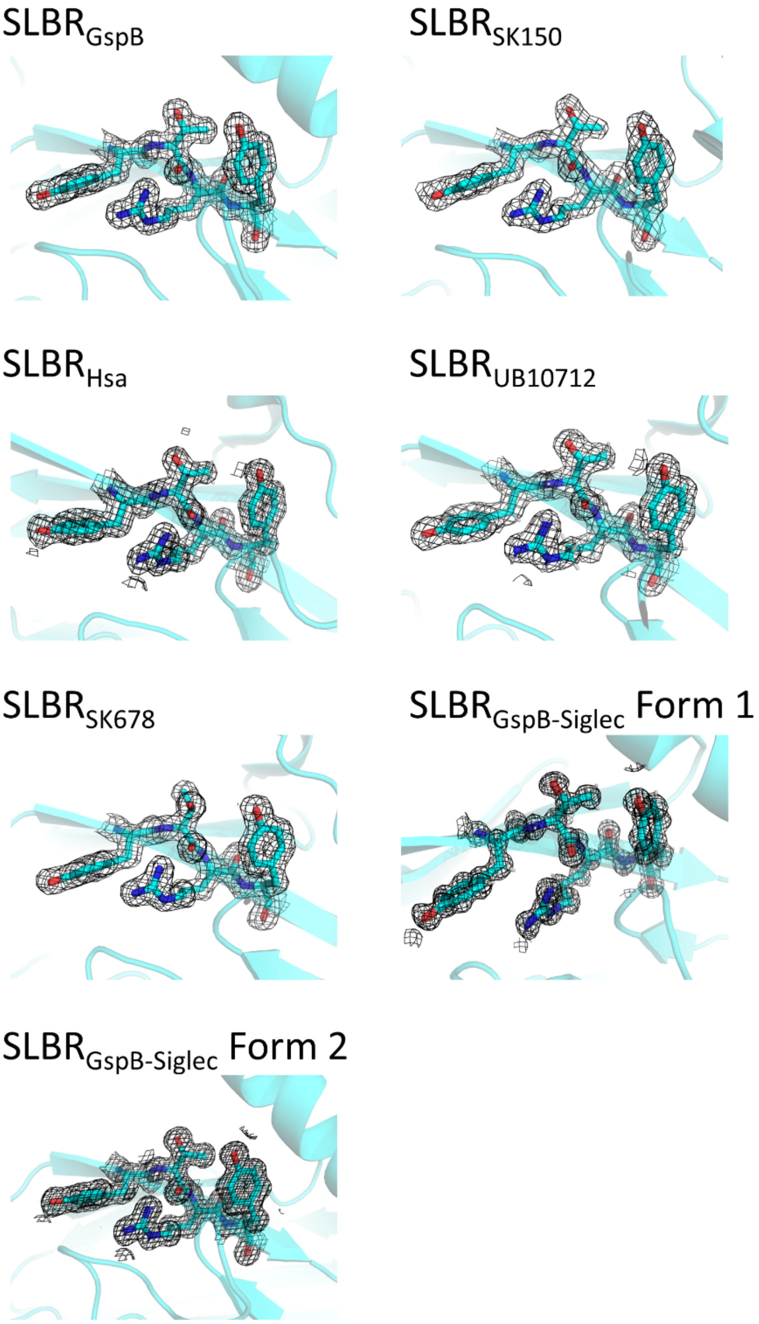

Supplementary Figure 22. Representative electron density for the ϕTR*x* sialic acid-binding motif in unliganded SLBRs. Each SLBR is shown as a cartoon representation. $2|F_o| - |F_c|$ electron density was calculated in Phenix[56] and is shown in grey mesh contoured at 1σ.

Supplementary References

1. Pyburn TM, *et al.* A structural model for binding of the serine-rich repeat adhesin GspB to host carbohydrate receptors. *PLoS pathogens* **7**, e1002112 (2011).

2. Bensing BA, *et al.* Structural Basis for Sialoglycan Binding by the Streptococcus sanguinis SrpA Adhesin. *The Journal of biological chemistry* **291**, 7230-7240 (2016).

3. Otwinowski Z, Minor W. Processing of X-ray Diffraction Data Collected in Oscillation Mode. In: *Methods in Enzymology* (eds Carter C, Jr, Sweet R). Academic Press (1997).

4. Kabsch W. Xds. *Acta Crystallogr D* **66**, 125-132 (2010).

5. Adams PD, *et al.* PHENIX: a comprehensive Python-based system for macromolecular structure solution. *Acta crystallographica Section D, Biological crystallography* **66**, 213-221 (2010).

6. Loukachevitch LV, *et al.* Structures of the Streptococcus sanguinis SrpA Binding Region with Human Sialoglycans Suggest Features of the Physiological Ligand. *Biochemistry* **55**, 5927-5937 (2016).

7. Stubbs HE, *et al.* Tandem sialoglycan-binding modules in a Streptococcus sanguinis serine-rich repeat adhesin create target dependent avidity effects. *The Journal of biological chemistry*, (2020).
