# Supplementary material for: Origins of glycan selectivity in streptococcal Siglec-like adhesins suggest mechanisms of receptor adaptation
Source: Nat Commun. 2022 May 18;13:2753. doi: 10.1038/s41467-022-30509-y (PMC9117288; doi:10.1038/s41467-022-30509-y)
Supplement: Supplementary file 3 — Description of Additional Supplementary Files [file 41467_2022_30509_MOESM3_ESM.docx]

**File Name: Supplementary Data 1. Center for Functional Glycomics (CFG) glycan array version 5.4.** The table lists the glycans included in the CFG array version 5.4, which was used in this study.

**File Name: Supplementary Data 2. Mass spectrometry in the 150 kD region of the blots identifies MUC7 as the major species.**  Proteins are listed by abundance, with MUC7 the most abundant protein in this region (n=2 samples from independent donors). Minor amounts of MUC5B (>10 mDa) and gp340 (a.k.a. DMBT1; 340 kDa) or fragments thereof, were also detected in the 140-160 kDa region, but the MUC5B has larger, more extensively branched less-sialylated *O*-glycans, and gp340 is primarily N-glycosylated. Remaining proteins lack *O-*linked glycosylations.

**File Name: Supplementary Data 3. Complete peptide results from mass spectrometry protein identification.** All identified peptides from the experiments that are presented in Supplementary Data 2.
