# Supplementary material for: Origins of glycan selectivity in streptococcal Siglec-like adhesins suggest mechanisms of receptor adaptation
Source: Nat Commun. 2022 May 18;13:2753. doi: 10.1038/s41467-022-30509-y (PMC9117288; doi:10.1038/s41467-022-30509-y)
Supplement: Supplementary file 7 — Reporting Summary [file 41467_2022_30509_MOESM7_ESM.pdf]

nature portfolio

Corresponding author(s): TM Iverson
Last updated by author(s): Jan 31, 2022

Reporting Summary

Nature Portfolio wishes to improve the reproducibility of the work that we publish. This form provides structure for consistency and transparency in reporting. For further information on Nature Portfolio policies, see our Editorial Policies and the Editorial Policy Checklist.

Statistics

For all statistical analyses, confirm that the following items are present in the figure legend, table legend, main text, or Methods section.

n/a	Confirmed	
☐	☒	The exact sample size (n) for each experimental group/condition, given as a discrete number and unit of measurement
☐	☒	A statement on whether measurements were taken from distinct samples or whether the same sample was measured repeatedly
☐	☒	The statistical test(s) used AND whether they are one- or two-sided<br>*Only common tests should be described solely by name; describe more complex techniques in the Methods section.*
☒	☐	A description of all covariates tested
☒	☐	A description of any assumptions or corrections, such as tests of normality and adjustment for multiple comparisons
☐	☒	A full description of the statistical parameters including central tendency (e.g. means) or other basic estimates (e.g. regression coefficient) AND variation (e.g. standard deviation) or associated estimates of uncertainty (e.g. confidence intervals)
☐	☒	For null hypothesis testing, the test statistic (e.g. F, t, r) with confidence intervals, effect sizes, degrees of freedom and P value noted<br>*Give P values as exact values whenever suitable.*
☒	☐	For Bayesian analysis, information on the choice of priors and Markov chain Monte Carlo settings
☒	☐	For hierarchical and complex designs, identification of the appropriate level for tests and full reporting of outcomes
☒	☐	Estimates of effect sizes (e.g. Cohen's d, Pearson's r), indicating how they were calculated

Our web collection on statistics for biologists contains articles on many of the points above.

Software and code

Policy information about availability of computer code

Data collection	For x-ray diffraction data, data collection used software available through the X-ray generator (Bruker, SC-XRD) or at the beamline (Blu-Ice, remote access). Binding data did not use software for data collection. Data collection software including version numbers is listed in the supplemental tables describing data collection.
Data analysis	Prism 9.3.0 was used for statistical analysis. For diffraction data processing and analysis, we used XDS (version June 1, 2017), HKL2000 v 712, COOT v 0.9, and PHENIX 1.18.2. The Phaser subroutine is within phenix and does not have a separate version number. Program version numbers are now included in the materials and methods in the main text and/or in the supplementary information in legends to the data collection tables, as appropriate.

For manuscripts utilizing custom algorithms or software that are central to the research but not yet described in published literature, software must be made available to editors and reviewers. We strongly encourage code deposition in a community repository (e.g. GitHub). See the Nature Portfolio guidelines for submitting code & software for further information.

Data

Policy information about availability of data

All manuscripts must include a data availability statement. This statement should provide the following information, where applicable:

- Accession codes, unique identifiers, or web links for publicly available datasets
- A description of any restrictions on data availability
- For clinical datasets or third party data, please ensure that the statement adheres to our policy

Source data are provided with this paper, consisting of raw data for ELISAs and uncropped gels. Atomic coordinates and structure factors have been deposited into the RCSB Protein Data Bank at www.rcsb.org and may be accessed via accession codes or direct hypoerlink to the DOI. The codes are 6EFA (doi.org/10.2210/

pdb6EFA/pdb), 6EFB (doi.org/10.2210/pdb6EFB/pdb), 6EFC (doi.org/10.2210/pdb6EFC/pdb), 6EFD (doi.org/10.2210/pdb6EFD/pdb), 6EFF (doi.org/10.2210/pdb6EFF/pdb), 6EFI (doi.org/10.2210/pdb6EFI/pdb), 6EF7 (doi.org/10.2210/pdb6EF7/pdb), 6EF9 (doi.org/10.2210/pdb6EF9/pdb), 6X3Q (doi.org/10.2210/pdb6X3Q/pdb), 6X3K (doi.org/10.2210/pdb6X3K/pdb), 7KMJ (doi.org/10.2210/pdb7KMJ/pdb). Raw X-ray diffraction data have been deposited into SBGrid (data.sbgrid.org) with accession codes and direct hyperlinks of 328 (doi.org/10.15785/SBGRID/328), 329 (doi.org/10.15785/SBGRID/329), 507 (doi.org/10.15785/SBGRID/507), 508 (doi.org/10.15785/SBGRID/508), 509 (doi.org/10.15785/SBGRID/509), 510 (doi.org/10.15785/SBGRID/510), 601 (doi.org/10.15785/SBGRID/601), 604 (doi.org/10.15785/SBGRID/604), 787 (doi.org/10.15785/SBGRID/787), 788 (doi.org/10.15785/SBGRID/788), 812 (doi.org/10.15785/SBGRID/812), 813 (doi.org/10.15785/SBGRID/813). A summary of these accession codes is also listed in Tables S1, S2, and S3. Glycomic data were deposited in MassIVE (https://massive.ucsd.edu/) with the dataset identifier MSV000088327 and direct hyperlink doi.org/10.25345/C5G577.

Field-specific reporting

Please select the one below that is the best fit for your research. If you are not sure, read the appropriate sections before making your selection.

☒ Life sciences ☐ Behavioural & social sciences ☐ Ecological, evolutionary & environmental sciences

For a reference copy of the document with all sections, see nature.com/documents/nr-reporting-summary-flat.pdf

Life sciences study design

All studies must disclose on these points even when the disclosure is negative.

Sample size	sample size of n=3 was used as the minimum number to allow for the calculation of standard deviation. It was not pre-determined by statistical methods.
Data exclusions	no data were excluded from the biophysical analyses.
Replication	all experiments were performed with a minimum of three independent measurements and all replicates were successful
Randomization	not applicable - biological samples were given numeric identifiers. ELISAs were measured in a logical concentration series.
Blinding	biological samples were given numeric identifiers.

Reporting for specific materials, systems and methods

We require information from authors about some types of materials, experimental systems and methods used in many studies. Here, indicate whether each material, system or method listed is relevant to your study. If you are not sure if a list item applies to your research, read the appropriate section before selecting a response.

Materials & experimental systems

n/a	Involved in the study	
☐	☒	Antibodies
☒	☐	Eukaryotic cell lines
☒	☐	Palaeontology and archaeology
☒	☐	Animals and other organisms
☐	☒	Human research participants
☒	☐	Clinical data
☒	☐	Dual use research of concern

Methods

n/a	Involved in the study	
☒	☐	ChIP-seq
☒	☐	Flow cytometry
☒	☐	MRI-based neuroimaging

Antibodies

Antibodies used	Anti-GST antibody was from Invitrogen (A5800) was used at a 1:500 dilution. Peroxidase-conjugated goat anti-rabbit IgG was from Sigma (A0545) and used at a 1:5,000 dilution
Validation	The anti-GST antibody recognizes the GST affinity tag and is broadly used. The ThermoFisher web site provides 67 references demonstrating its validation; three of these references are for ELISA, the technique used here. See https://www.thermofisher.com/antibody/product/GST-Tag-Antibody-Polyclonal/A-5800. The goat anti-rabbit IgG recognizes rabbit IgG and is similarly widely-used. The Sigma web site points to 1,348 papers that use this antibody.

Human research participants

Policy information about studies involving human research participants

Population characteristics	Salivary samples were a from a banked collection that we accessed for free. We did not collect samples. This is an older collection and we do not have access to demographic information for the donors.
Recruitment	We did not recruit participants.
Ethics oversight	De-identified samples of banked SMSL saliva were provided by S. Fisher (UCSF). Because these specimens were de-identified prior to gifting, our use of this material was exempt from approval by the UCSF Institutional Review Board. This is now stated in the methods.

Note that full information on the approval of the study protocol must also be provided in the manuscript.
