# Supplementary material for: Origins of glycan selectivity in streptococcal Siglec-like adhesins suggest mechanisms of receptor adaptation
Source: Nat Commun. 2022 May 18;13:2753. doi: 10.1038/s41467-022-30509-y (PMC9117288; doi:10.1038/s41467-022-30509-y)
Supplement: Supplementary file 8 — Source Data [file 41467_2022_30509_MOESM8_ESM.zip › source_data/raw-blots/raw-blots-labeled.pptx]

## Slide 1
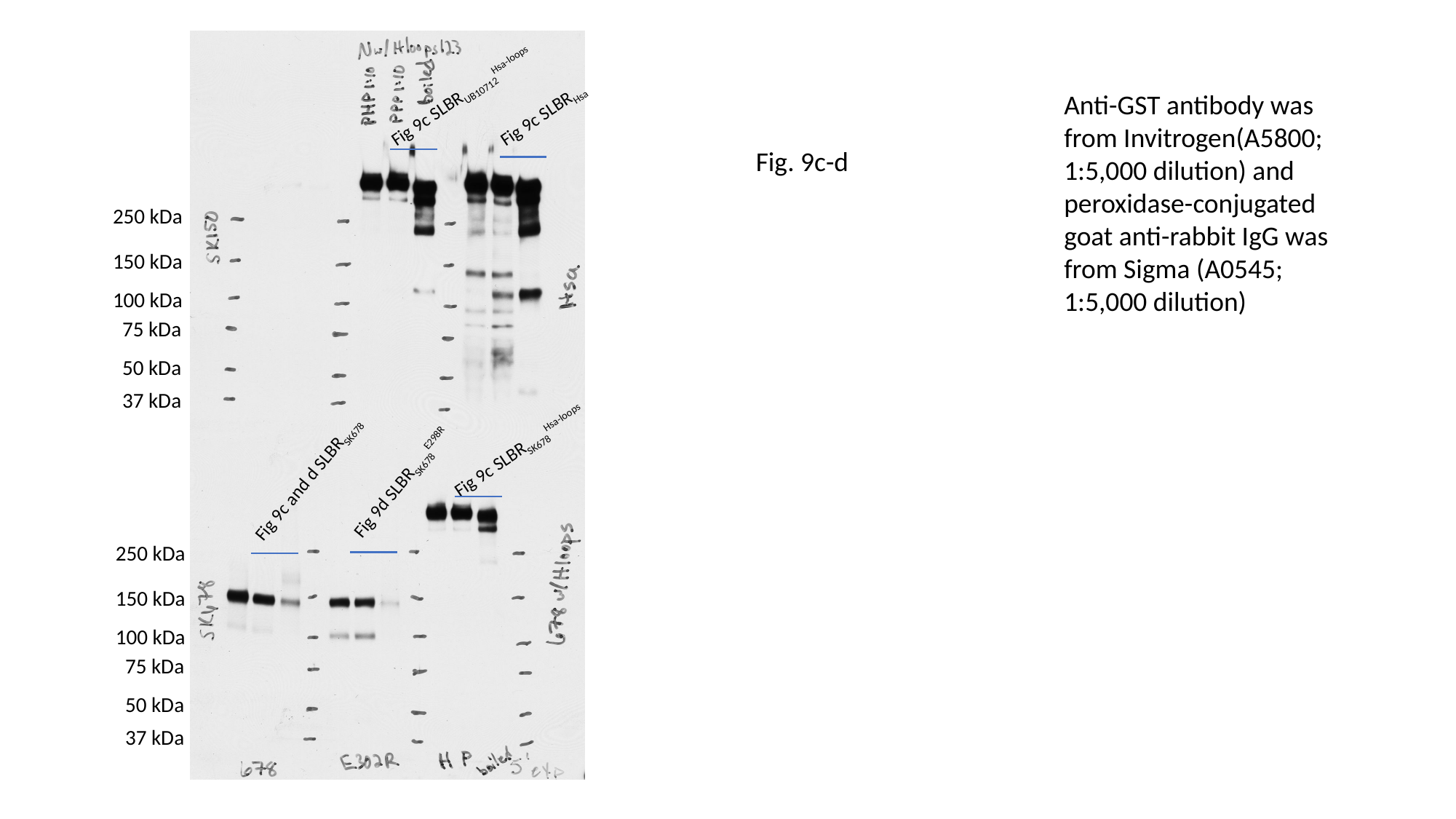

Fig 9c SLBRUB10712Hsa-loops
Anti-GST antibody was from Invitrogen(A5800; 1:5,000 dilution) and peroxidase-conjugated goat anti-rabbit IgG was from Sigma (A0545; 1:5,000 dilution)
Fig 9c SLBRHsa
Fig. 9c-d
250 kDa
150 kDa
100 kDa
 75 kDa
 50 kDa
 37 kDa
Fig 9c SLBRSK678Hsa-loops
Fig 9c and d SLBRSK678
Fig 9d SLBRSK678E298R
250 kDa
150 kDa
100 kDa
 75 kDa
 50 kDa
 37 kDa

## Slide 2
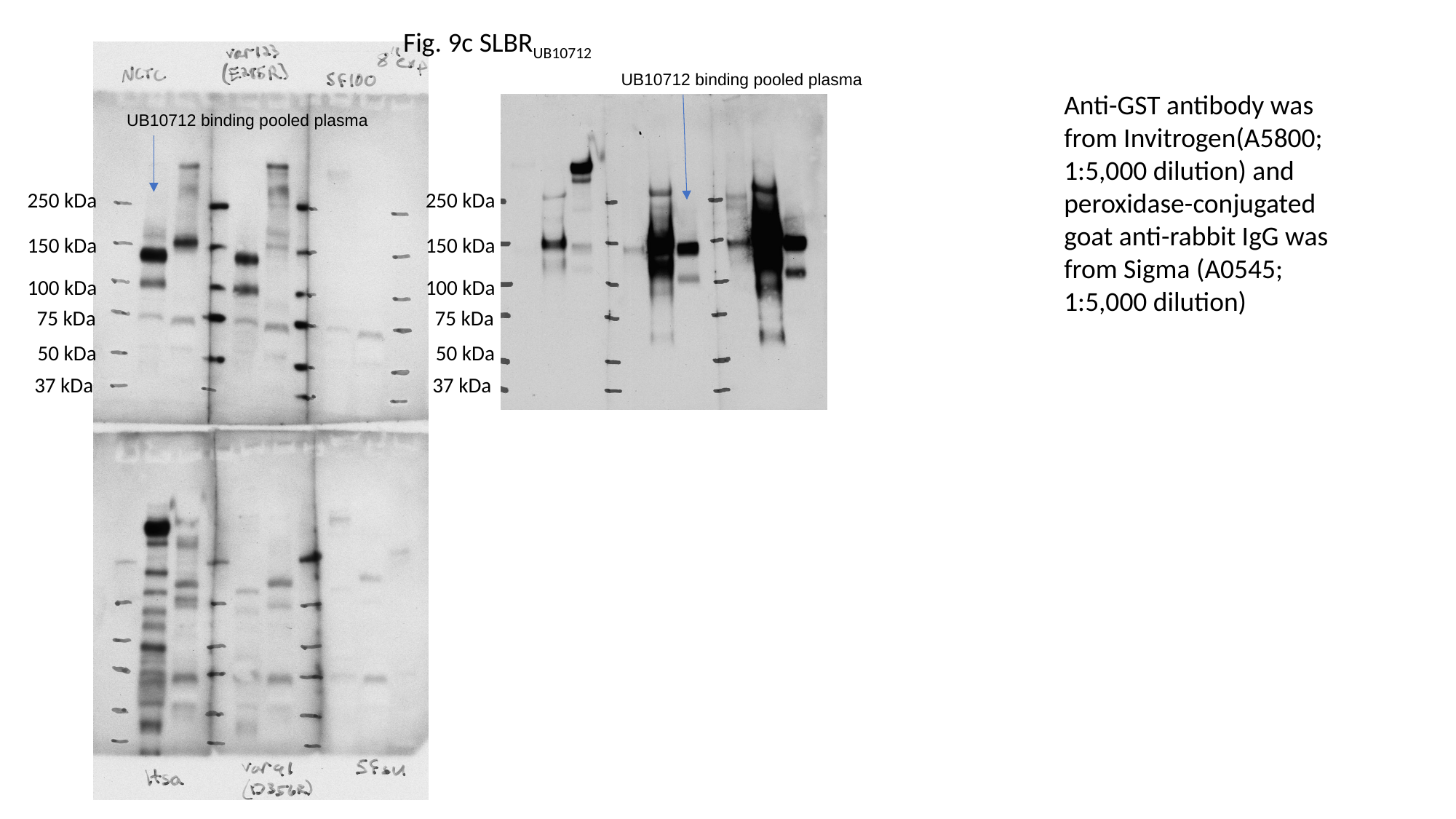

Fig. 9c SLBRUB10712
UB10712 binding pooled plasma
Anti-GST antibody was from Invitrogen(A5800; 1:5,000 dilution) and peroxidase-conjugated goat anti-rabbit IgG was from Sigma (A0545; 1:5,000 dilution)
UB10712 binding pooled plasma
250 kDa
250 kDa
150 kDa
150 kDa
100 kDa
100 kDa
 75 kDa
 75 kDa
 50 kDa
 50 kDa
 37 kDa
 37 kDa

## Slide 3
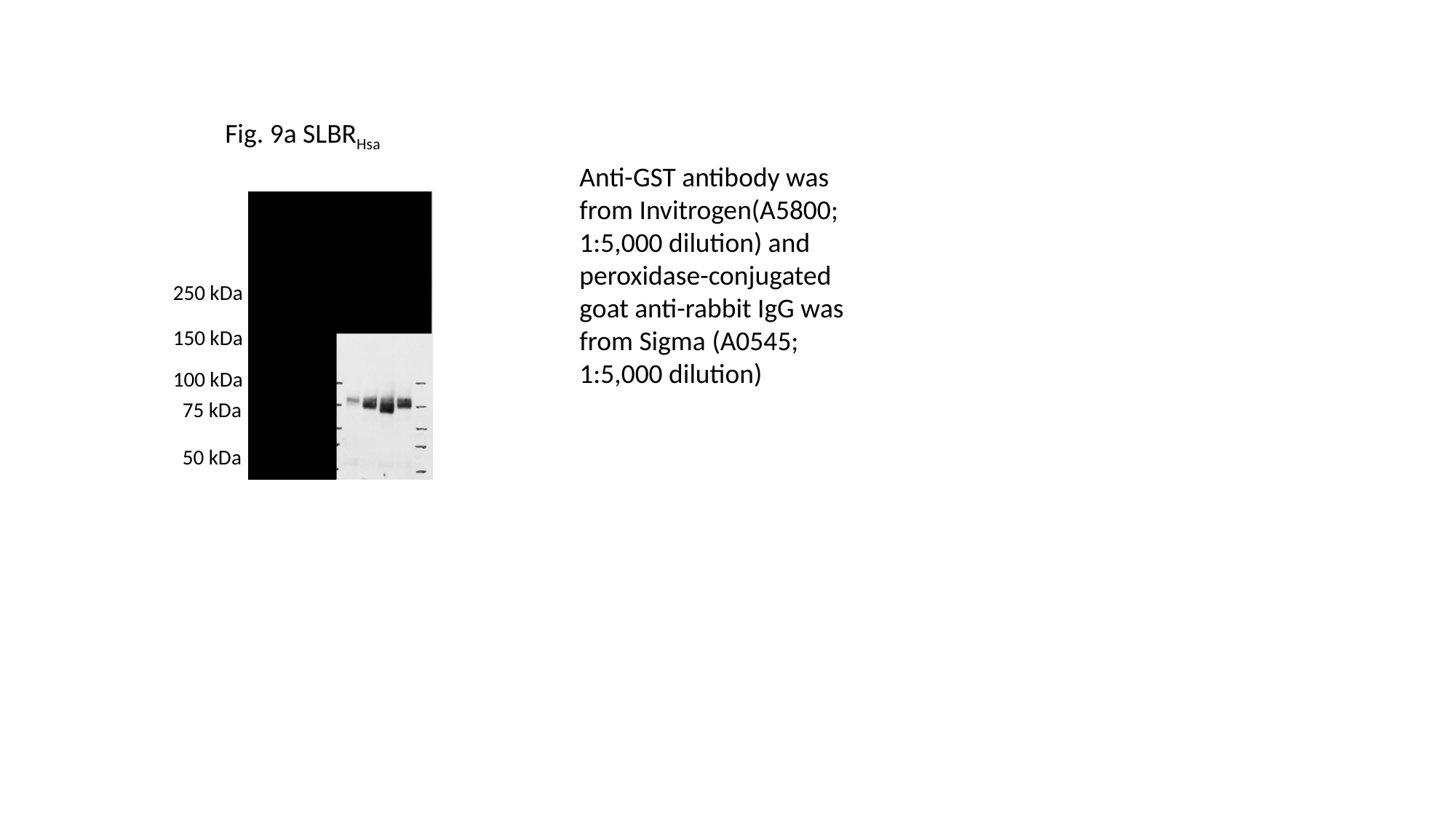

Fig. 9a SLBRHsa
Anti-GST antibody was from Invitrogen(A5800; 1:5,000 dilution) and peroxidase-conjugated goat anti-rabbit IgG was from Sigma (A0545; 1:5,000 dilution)
250 kDa
150 kDa
100 kDa
 75 kDa
 50 kDa

## Slide 4
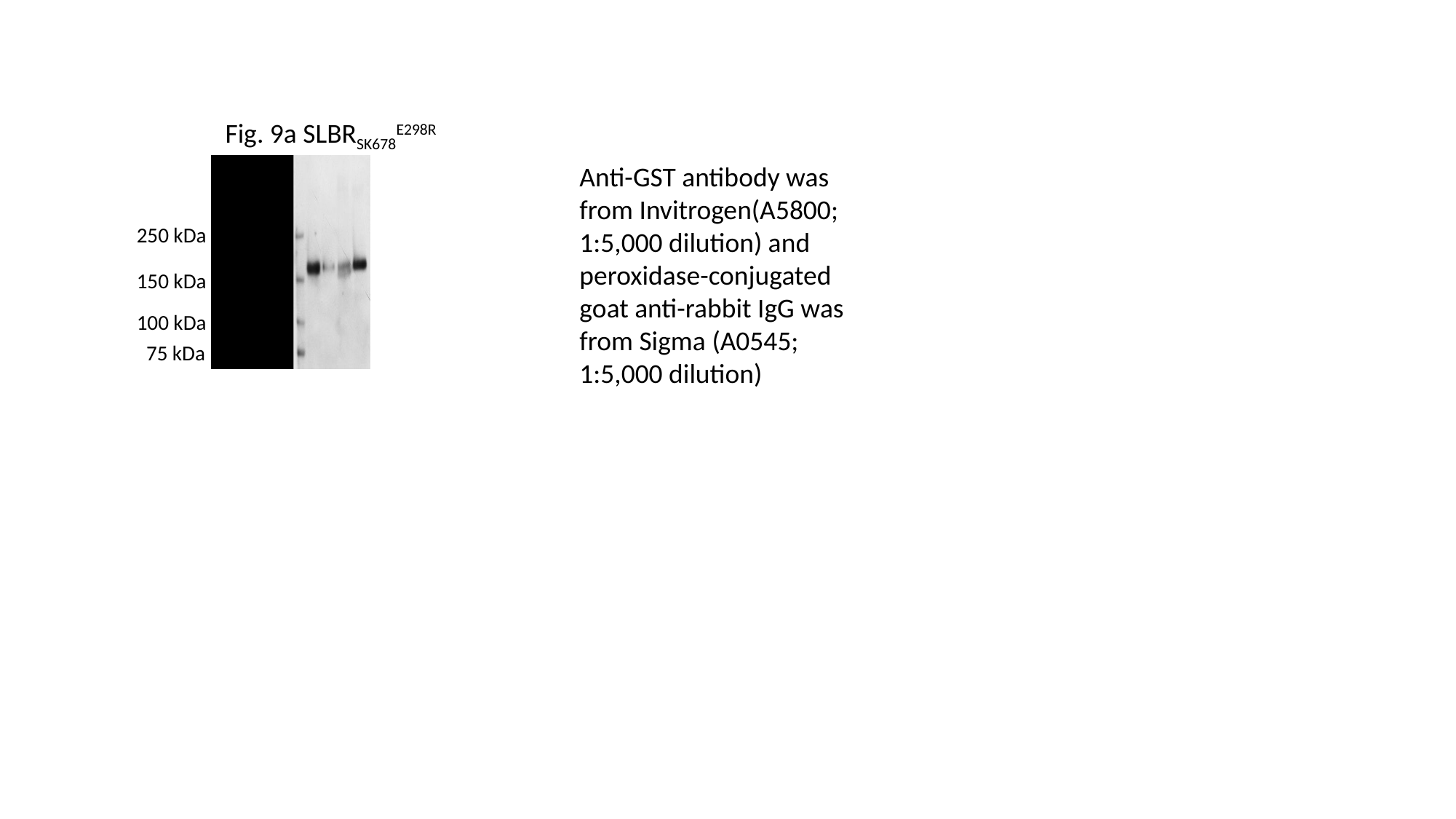

Fig. 9a SLBRSK678E298R
Anti-GST antibody was from Invitrogen(A5800; 1:5,000 dilution) and peroxidase-conjugated goat anti-rabbit IgG was from Sigma (A0545; 1:5,000 dilution)
250 kDa
150 kDa
100 kDa
 75 kDa

## Slide 5
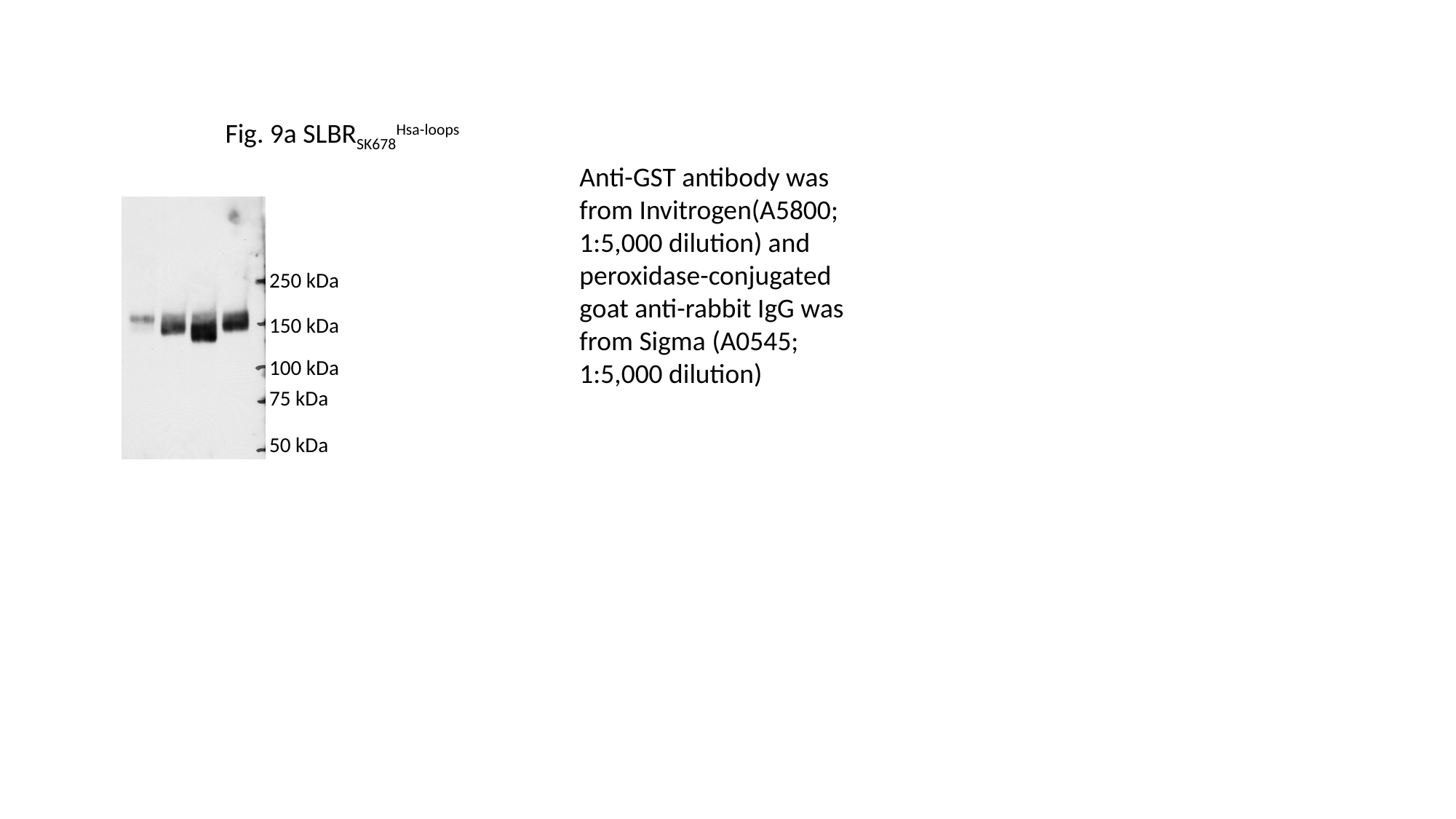

Fig. 9a SLBRSK678Hsa-loops
Anti-GST antibody was from Invitrogen(A5800; 1:5,000 dilution) and peroxidase-conjugated goat anti-rabbit IgG was from Sigma (A0545; 1:5,000 dilution)
250 kDa
150 kDa
100 kDa
75 kDa
50 kDa

## Slide 6
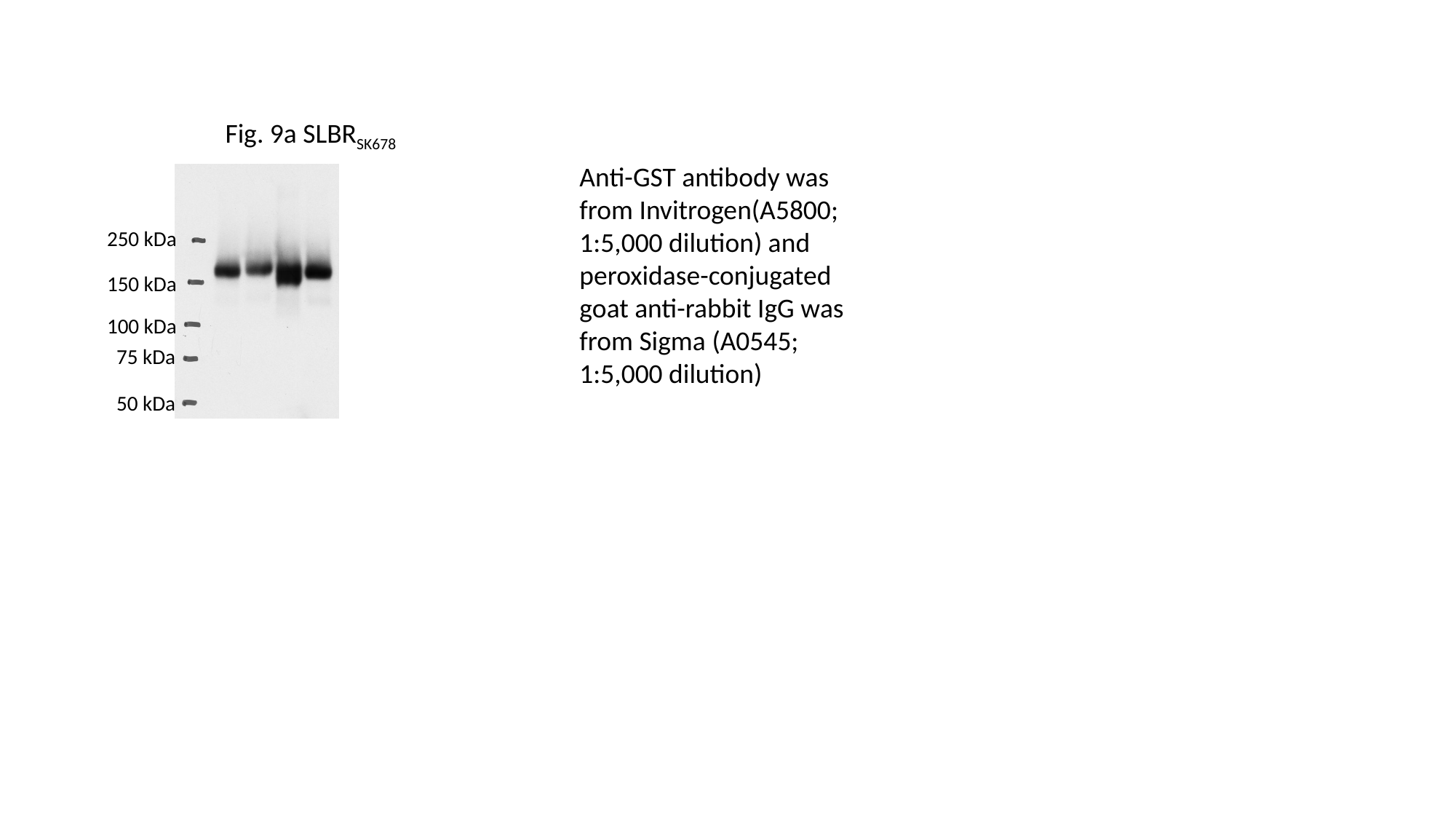

Fig. 9a SLBRSK678
Anti-GST antibody was from Invitrogen(A5800; 1:5,000 dilution) and peroxidase-conjugated goat anti-rabbit IgG was from Sigma (A0545; 1:5,000 dilution)
250 kDa
150 kDa
100 kDa
 75 kDa
 50 kDa

## Slide 7
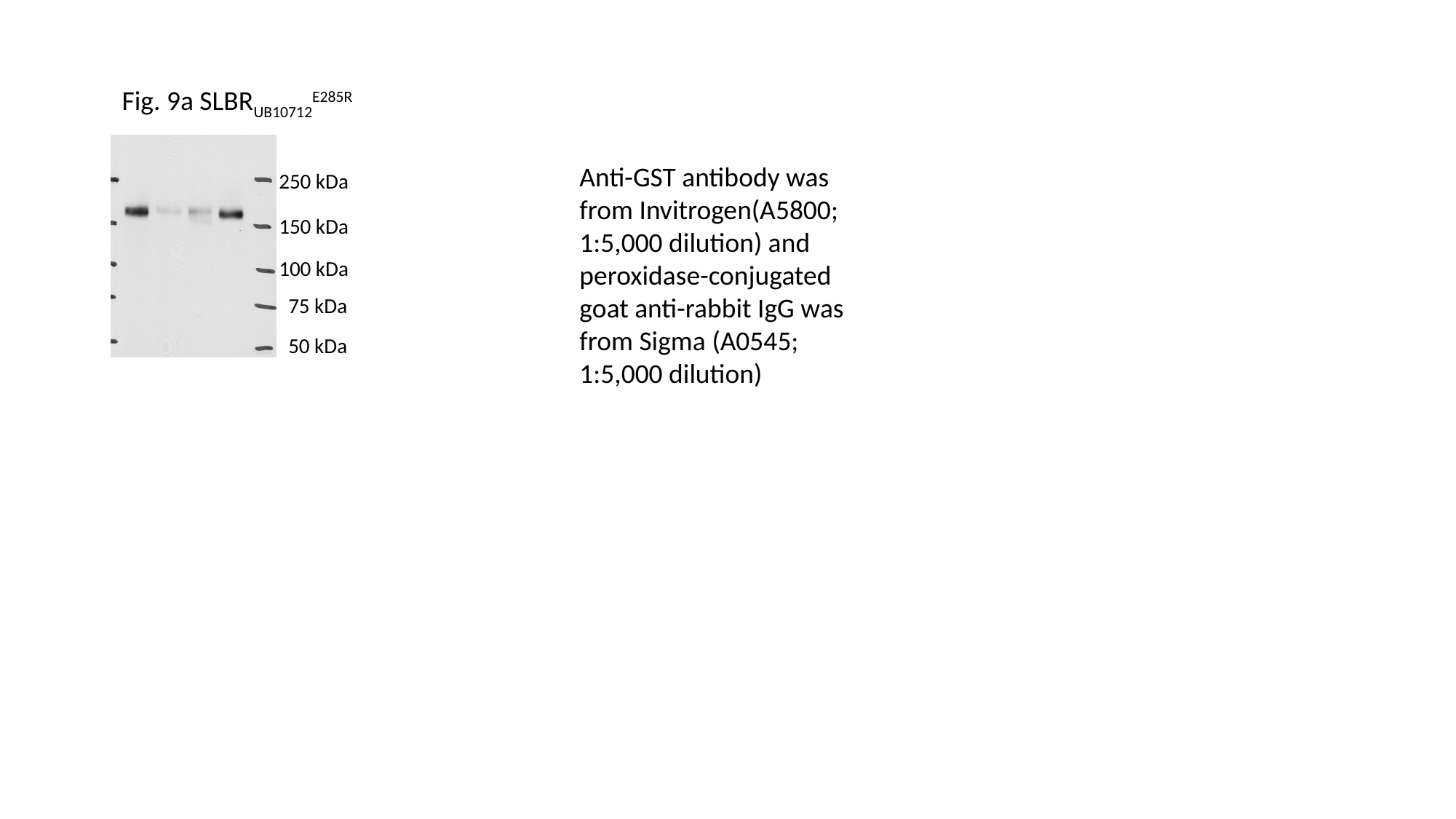

Fig. 9a SLBRUB10712E285R
Anti-GST antibody was from Invitrogen(A5800; 1:5,000 dilution) and peroxidase-conjugated goat anti-rabbit IgG was from Sigma (A0545; 1:5,000 dilution)
250 kDa
150 kDa
100 kDa
 75 kDa
 50 kDa

## Slide 8
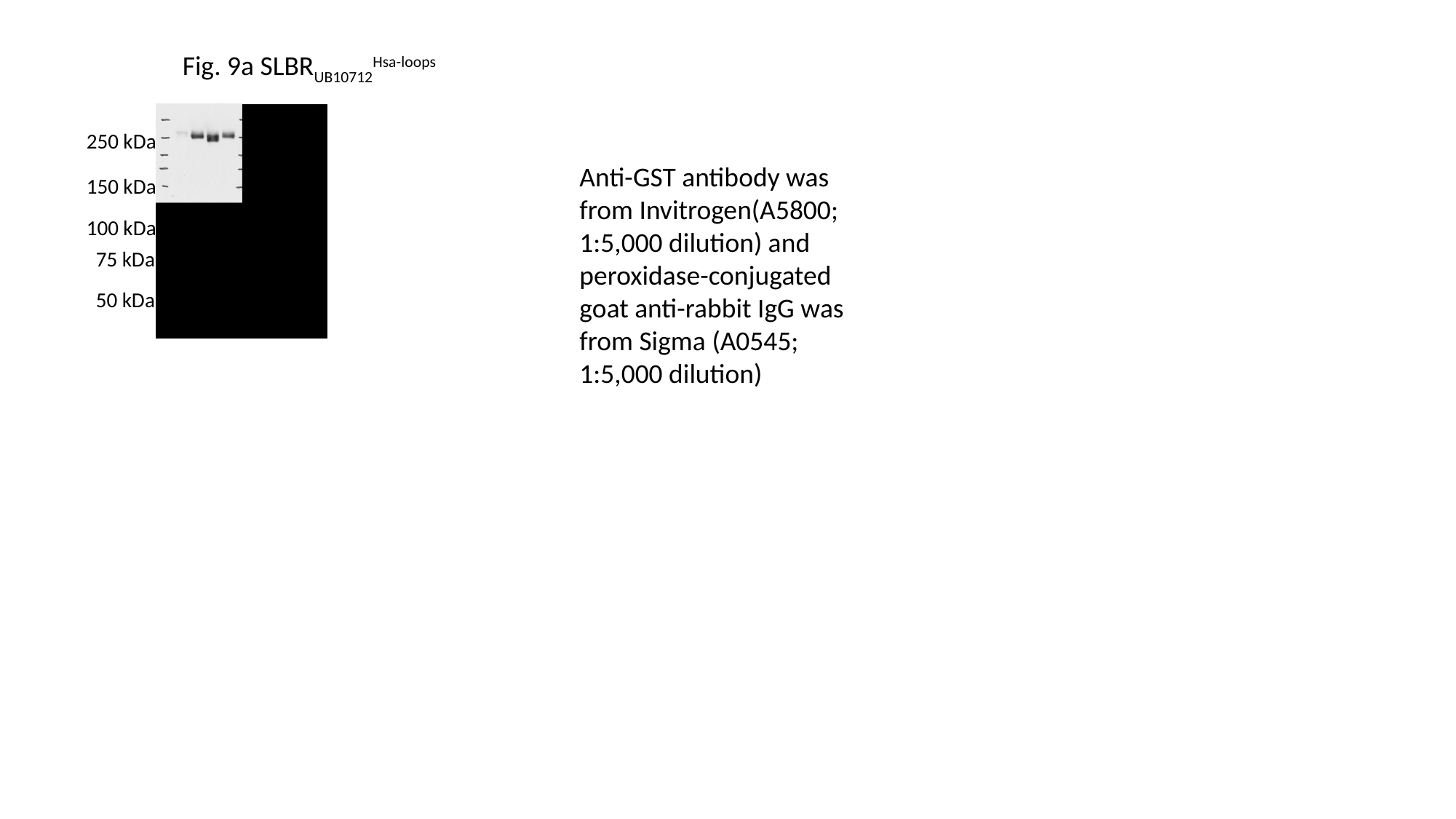

Fig. 9a SLBRUB10712Hsa-loops
250 kDa
Anti-GST antibody was from Invitrogen(A5800; 1:5,000 dilution) and peroxidase-conjugated goat anti-rabbit IgG was from Sigma (A0545; 1:5,000 dilution)
150 kDa
100 kDa
 75 kDa
 50 kDa

## Slide 9
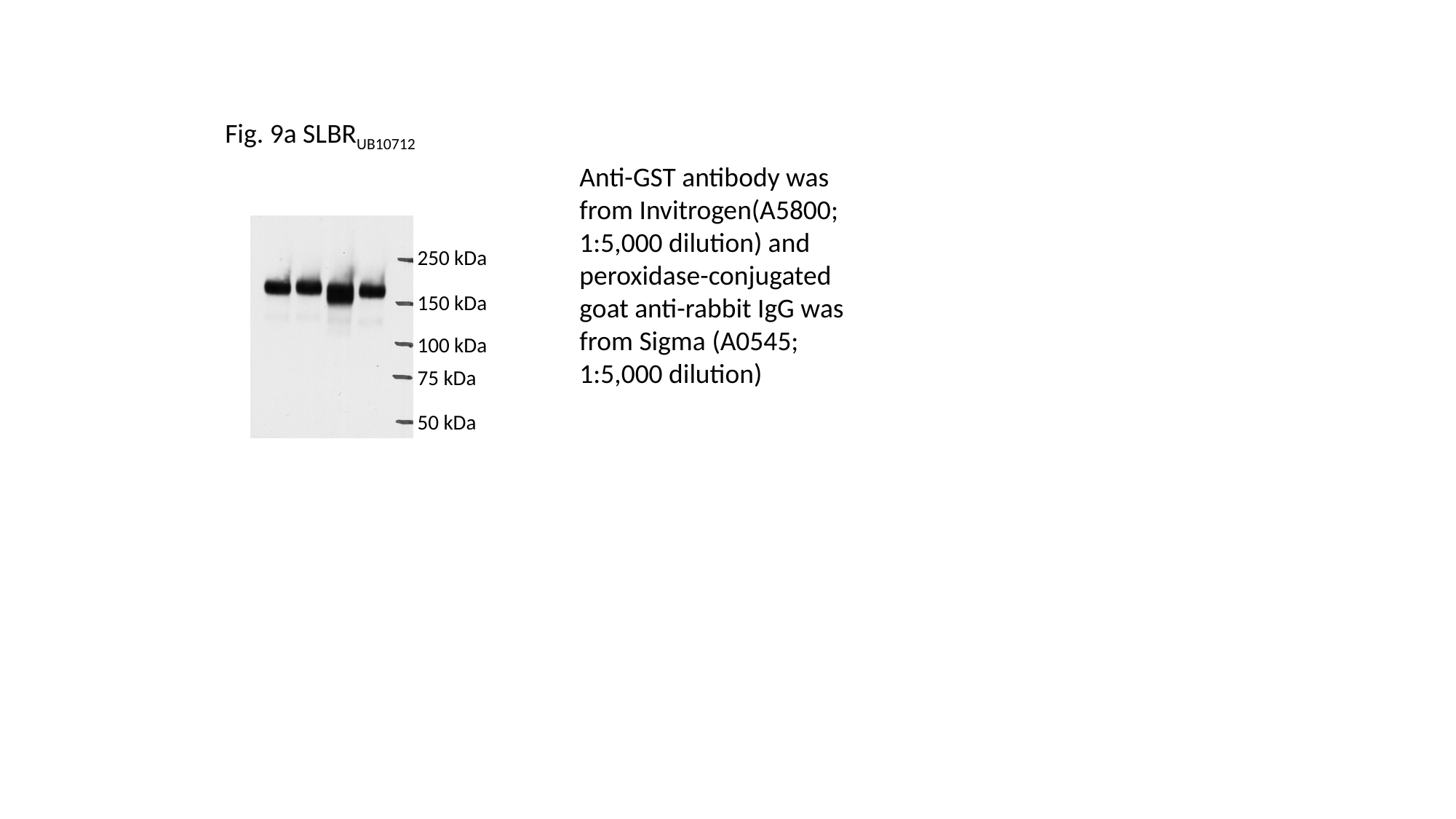

Fig. 9a SLBRUB10712
Anti-GST antibody was from Invitrogen(A5800; 1:5,000 dilution) and peroxidase-conjugated goat anti-rabbit IgG was from Sigma (A0545; 1:5,000 dilution)
250 kDa
150 kDa
100 kDa
75 kDa
50 kDa
