# Supplementary figures and images for: Origins of glycan selectivity in streptococcal Siglec-like adhesins suggest mechanisms of receptor adaptation
Source: Nat Commun. 2022 May 18;13:2753. doi: 10.1038/s41467-022-30509-y (PMC9117288; doi:10.1038/s41467-022-30509-y)

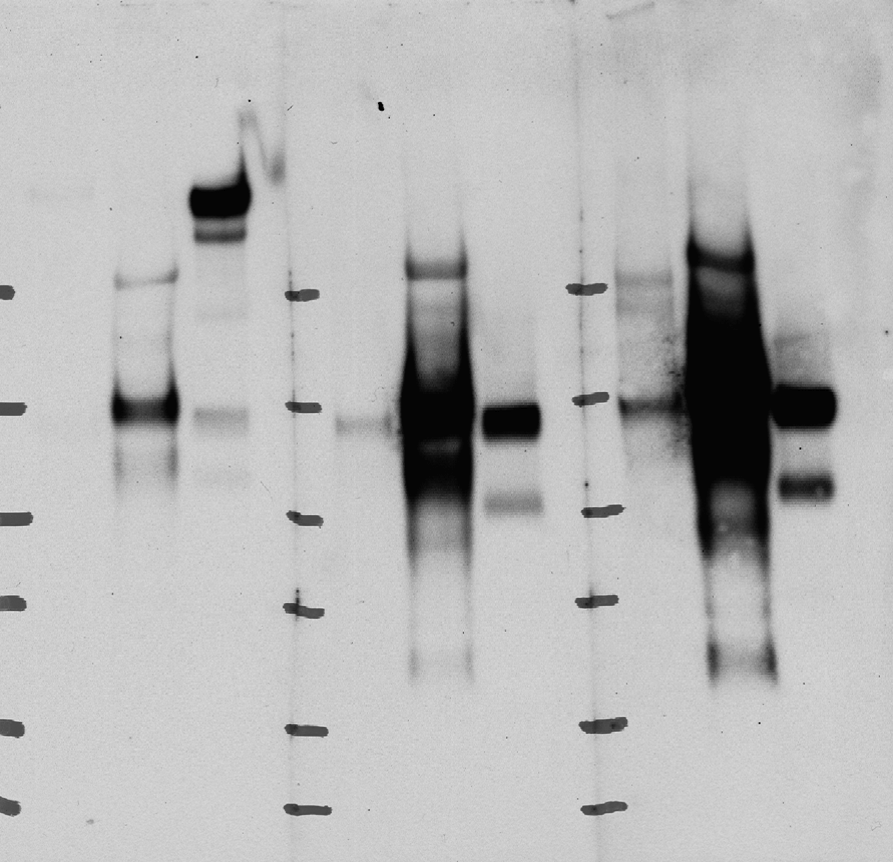

Supplement: Supplementary file 8 — Source Data [file 41467_2022_30509_MOESM8_ESM.zip › source_data/raw-blots/3BRsandpppC copy.tiff]

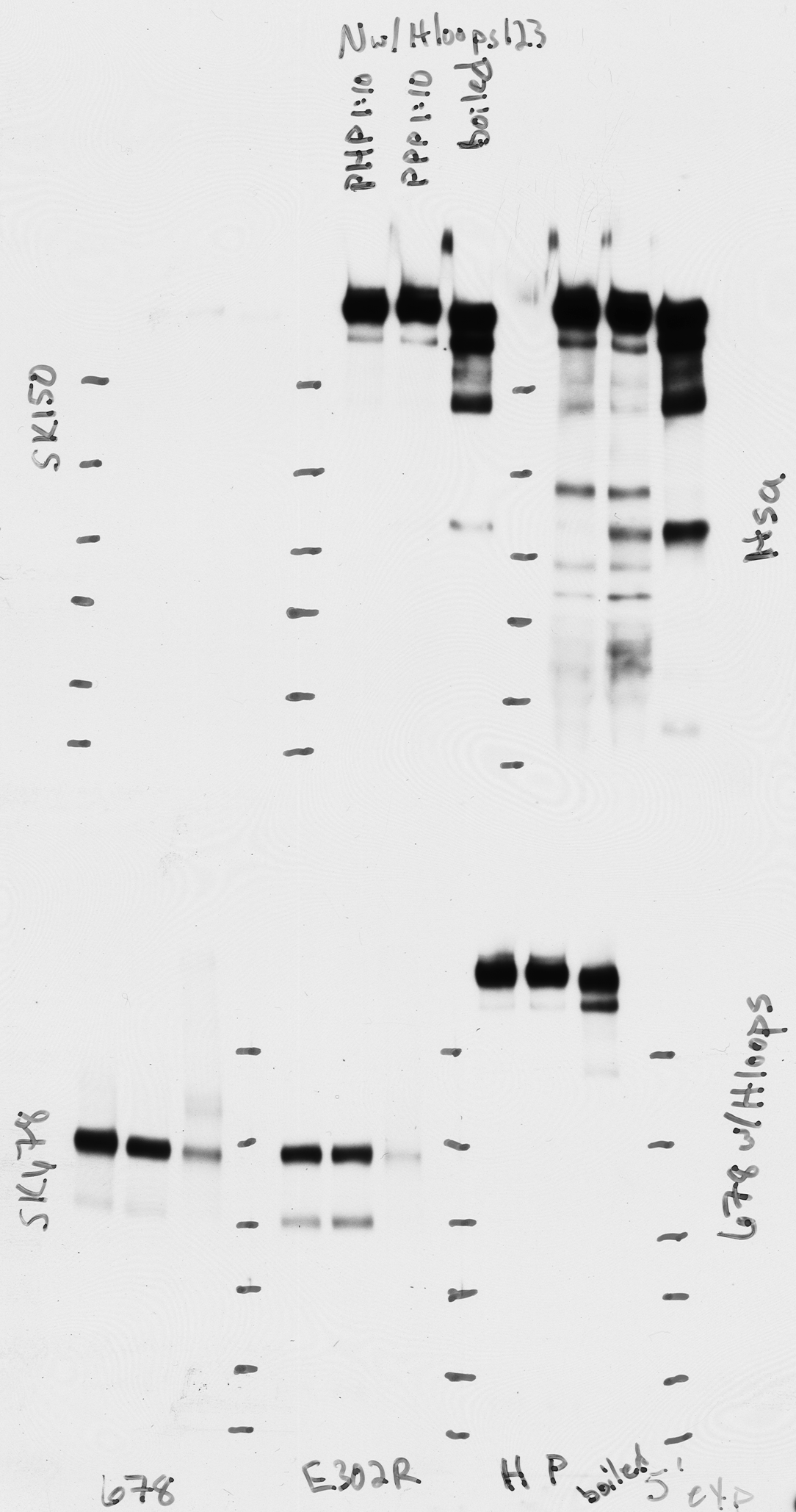

Supplement: Supplementary file 8 — Source Data [file 41467_2022_30509_MOESM8_ESM.zip › source_data/raw-blots/BRmutsMay2FWc.tiff]

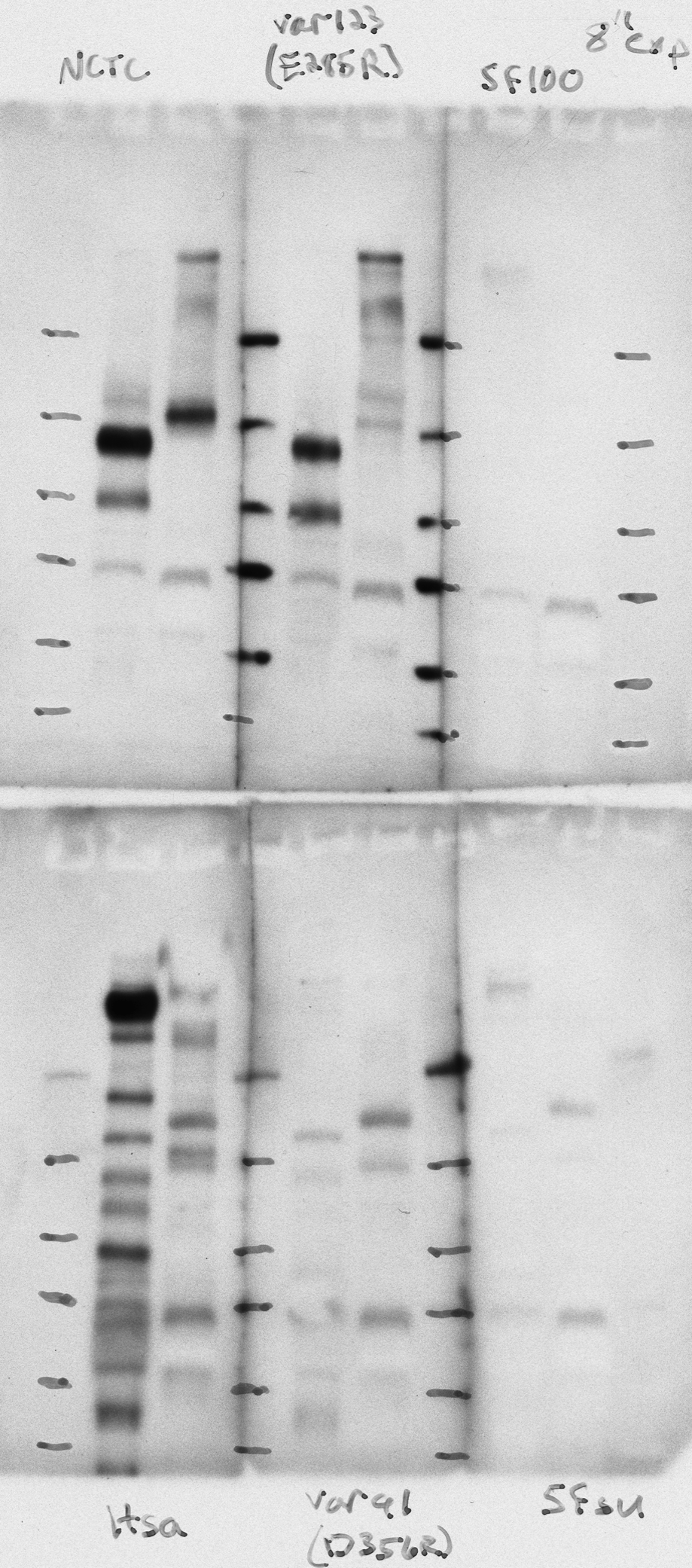

Supplement: Supplementary file 8 — Source Data [file 41467_2022_30509_MOESM8_ESM.zip › source_data/raw-blots/HandNmutsFWc copy.tiff]

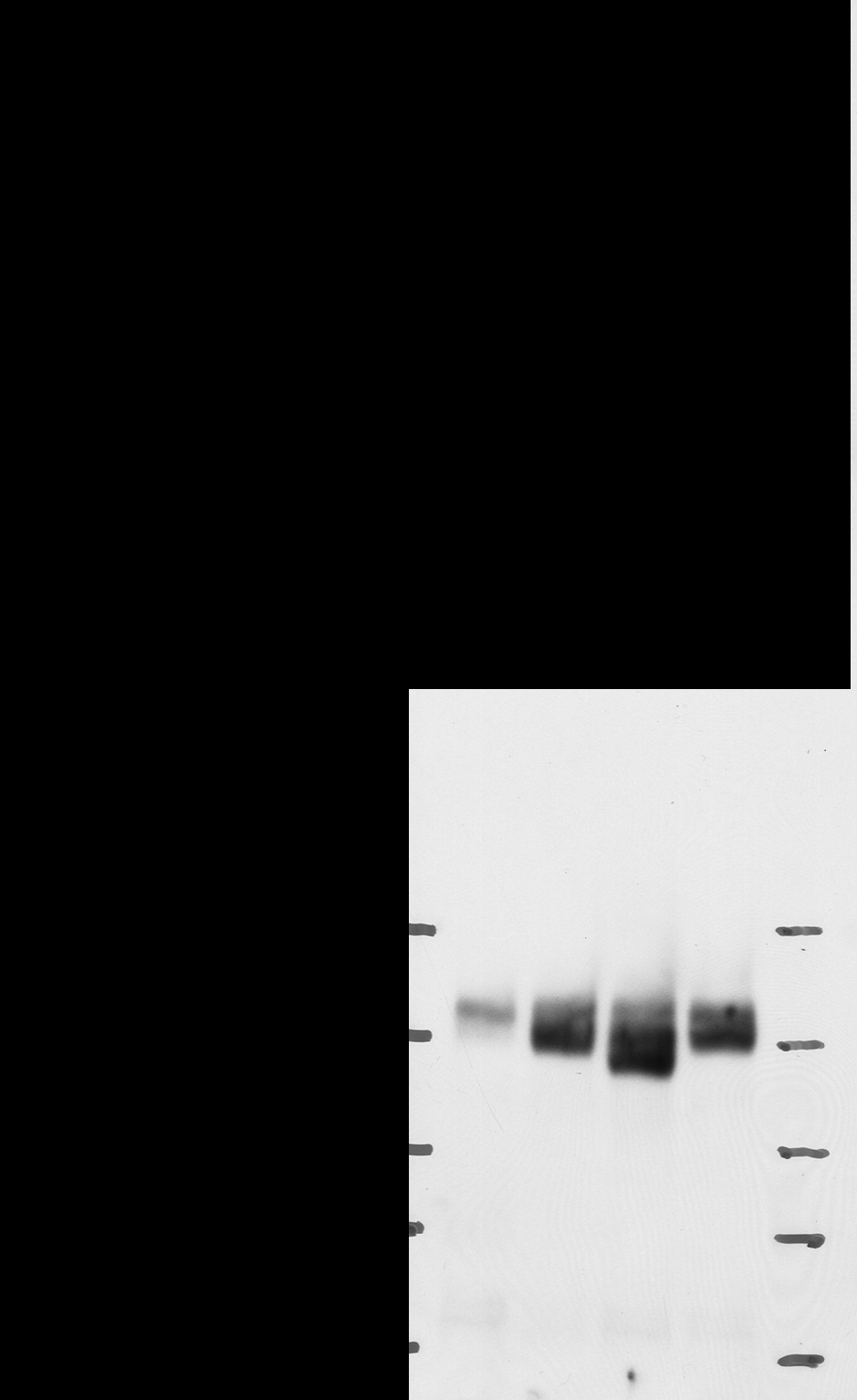

Supplement: Supplementary file 8 — Source Data [file 41467_2022_30509_MOESM8_ESM.zip › source_data/raw-blots/Hsa saliva.tiff]

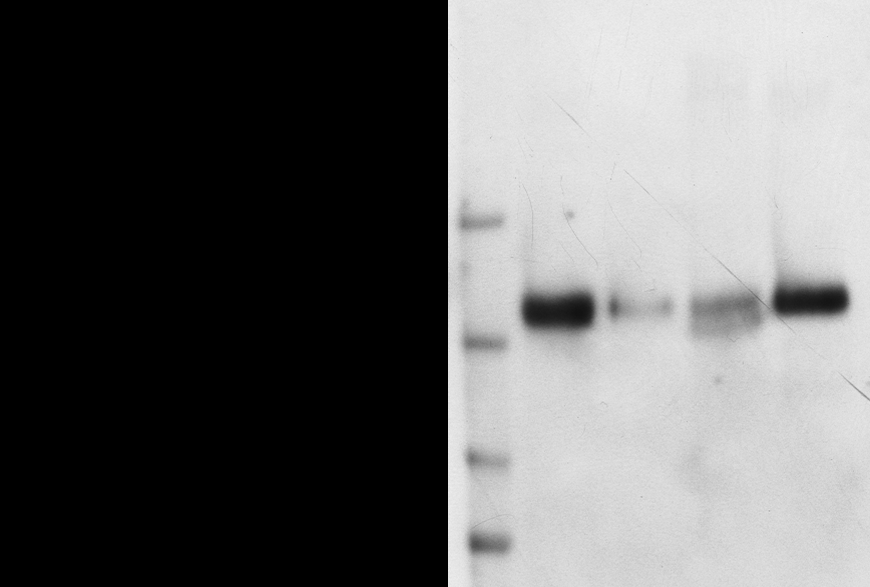

Supplement: Supplementary file 8 — Source Data [file 41467_2022_30509_MOESM8_ESM.zip › source_data/raw-blots/SK678 E298R saliva.tiff]

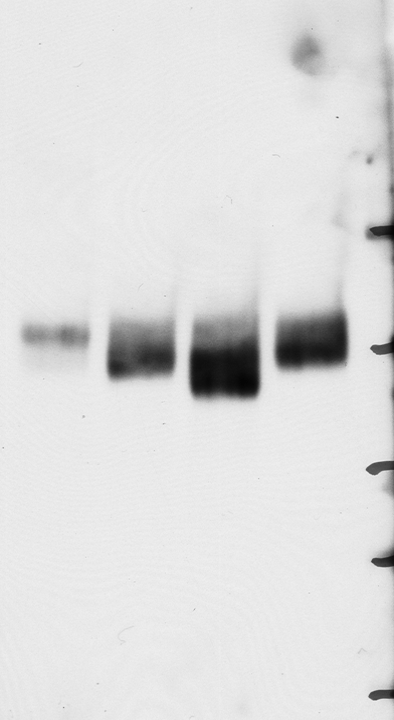

Supplement: Supplementary file 8 — Source Data [file 41467_2022_30509_MOESM8_ESM.zip › source_data/raw-blots/SK678 Hloops saliva.tiff]

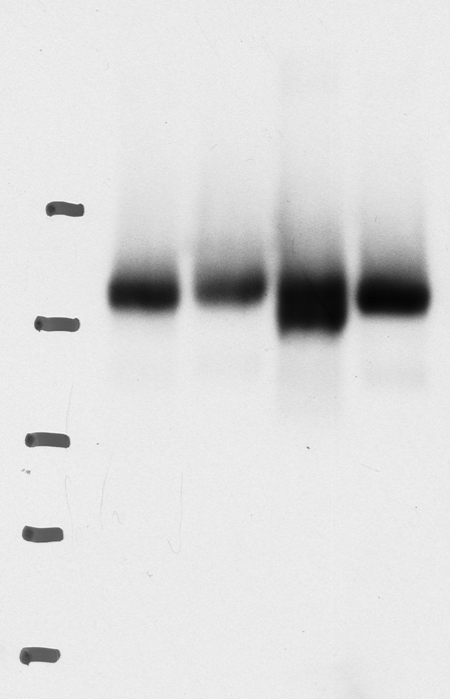

Supplement: Supplementary file 8 — Source Data [file 41467_2022_30509_MOESM8_ESM.zip › source_data/raw-blots/SK678 saliva.tiff]

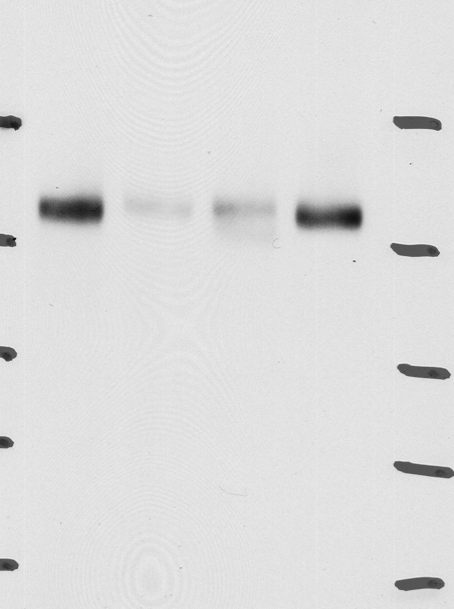

Supplement: Supplementary file 8 — Source Data [file 41467_2022_30509_MOESM8_ESM.zip › source_data/raw-blots/UB10712 E285R saliva.tiff]

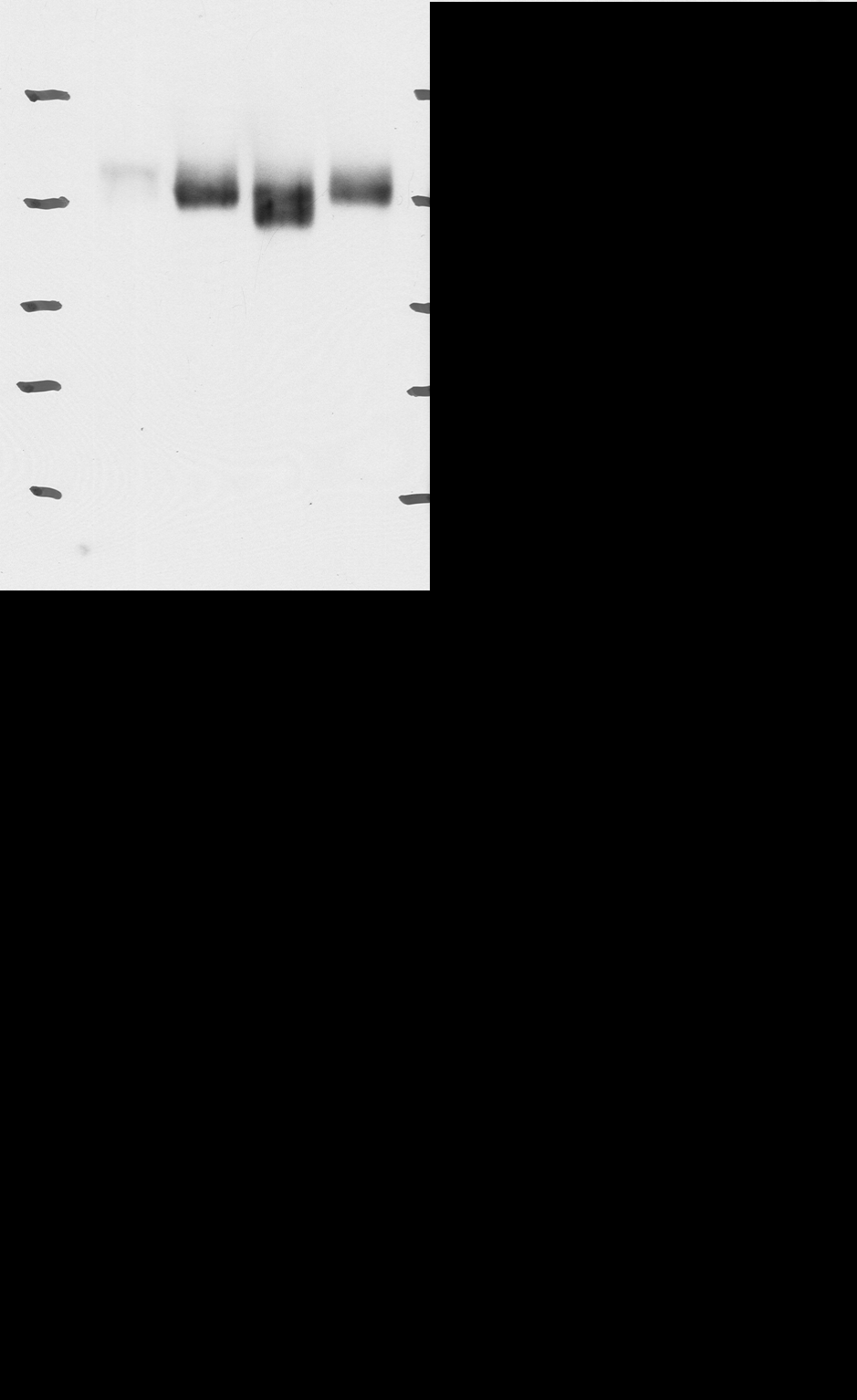

Supplement: Supplementary file 8 — Source Data [file 41467_2022_30509_MOESM8_ESM.zip › source_data/raw-blots/UB10712 Hloops saliva.tiff]

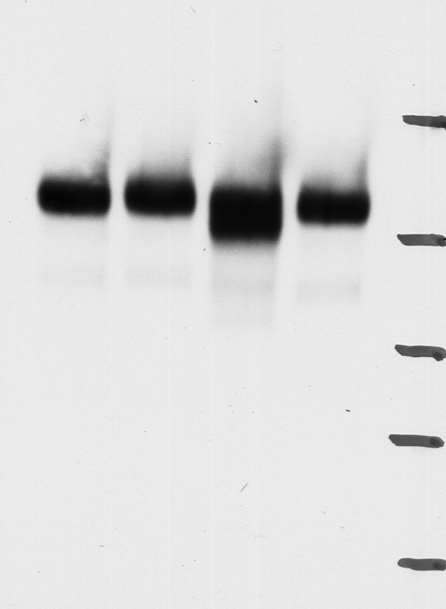

Supplement: Supplementary file 8 — Source Data [file 41467_2022_30509_MOESM8_ESM.zip › source_data/raw-blots/UB10712 saliva.tiff]
